# Flavonols modulate lateral root emergence by scavenging reactive oxygen species in *Arabidopsis thaliana*

**DOI:** 10.1074/jbc.RA120.014543

**Published:** 2020-12-25

**Authors:** Jordan M. Chapman, Gloria K. Muday

**Affiliations:** Biology Department, Wake Forest University, Winston Salem, North Carolina, USA

**Keywords:** flavonoid, *Arabidopsis thaliana*, ROS, antioxidant, redox signaling, lateral root development, CHI, chalcone isomerase, CHS, chalcone synthase, CM H_2_DCF-DA, 2′,7′-dichlorodihydro-fluorescein diacetate, DFR, dihydroflavonol 4-reductase, DHE, dihydroethidium, DIC, differential interference contrast, DPBA, diphenylboric acid 2-aminoethyl ester, FDA, fluorescein diacetate, F3H, flavonoid 3 hydroxylase, F3′H, flavonoid 3′hydroxylase, FLS1, flavonol synthase 1, IAA, indole-3-acetic acid, LC-MS, liquid chromatography–mass spectrometry, LRP, lateral root primordium, LSCM, laser scanning confocal microscopy, LUT, lookup table, OMT-1, O-methyl transferase 1, RBOH, respiratory burst oxidase homologs, ROS, reactive oxygen species

## Abstract

Flavonoids are a class of specialized metabolites with subclasses including flavonols and anthocyanins, which have unique properties as antioxidants. Flavonoids modulate plant development, but whether and how they impact lateral root development is unclear. We examined potential roles for flavonols in this process using *Arabidopsis thaliana* mutants with defects in genes encoding key enzymes in flavonoid biosynthesis. We observed the *tt4* and *fls1* mutants, which produce no flavonols, have increased lateral root emergence. The *tt4* root phenotype was reversed by genetic and chemical complementation. To more specifically define the flavonoids involved, we tested an array of flavonoid biosynthetic mutants, eliminating roles for anthocyanins and the flavonols quercetin and isorhamnetin in modulating lateral root development. Instead, two *tt7* mutant alleles, with defects in a branchpoint enzyme blocking quercetin biosynthesis, formed reduced numbers of lateral roots and *tt7-2* had elevated levels of kaempferol. Using a flavonol-specific dye, we observed that in the *tt7-2* mutant, kaempferol accumulated within lateral root primordia at higher levels than wild-type. These data are consistent with kaempferol, or downstream derivatives, acting as a negative regulator of lateral root emergence. We examined ROS accumulation using ROS-responsive probes and found reduced fluorescence of a superoxide-selective probe within the primordia of *tt7-2* compared with wild-type, but not in the *tt4* mutant, consistent with opposite effects of these mutants on lateral root emergence. These results support a model in which increased level of kaempferol in the lateral root primordia of *tt7*-2 reduces superoxide concentration and ROS-stimulated lateral root emergence.

Flavonoids are a class of plant specialized metabolites with important functions in modulating development and stress responses (1, 2). There are multiple subclasses of flavonoids including chalcones, flavones, isoflavonoids, flavanones, flavonols, and anthocyanins (3). The pathway begins with the conversion of 4-Coumaroyl CoA and Malonyl CoA to naringenin chalcone. Further downstream, dihydroflavonols are produced and can be converted to either flavonols or anthocyanins. Flavonols and anthocyanins are two of the best studied flavonoid subclasses due to their ubiquitous presence in multiple species, their diverse functionality (4), and antioxidant capacity (5, 6, 7, 8). *Arabidopsis thaliana* synthesizes three flavonols: kaempferol, quercetin, and isorhamnetin, which differ by the presence of a hydroxyl or methoxy group on their ring. The flavonoid biosynthesis pathway is well characterized in *Arabidopsis* with mutants isolated in the genes encoding each of the enzymes of the biosynthetic pathway (3).

Flavonoid biosynthetic mutants have been used to better understand the role of flavonols as signaling molecules that modulate development. The *Arabidopsis transparent testa 4* (*tt4*) mutant produces no flavonoids due to a mutation in the gene encoding the enzyme catalyzing the first committed step in the flavonoid biosynthesis pathway, chalcone synthase (CHS). Previous studies showed that *tt4* has increased root hair formation (9), ABA-induced stomatal closure (10), impaired gravity response (11), and greater sensitivity to environmental stress (12). Additional pathway mutants have been used to reveal which flavonols function in development, including identification of specific kaempferol derivatives that regulate leaf shape (13) and a role of quercetin in regulating gravitropic curvature and root hair initiation (9, 11). Using mutants impaired in flavonol biosynthesis in crop species, such as tomato, has also highlighted the role of flavonol metabolites in modulating environmentally responsive signaling pathways, such as ABA-dependent guard cell signaling that induces stomatal closure (14) and temperature-impaired pollen viability and pollen tube growth (15). Two mechanisms have been suggested to explain how flavonols regulate plant development. Flavonols function as negative regulators of auxin transport. *Arabidopsis* mutants with defects in flavonol synthesis have elevated levels of auxin transport (11, 16, 17, 18) and flavonols block auxin transport when transport proteins are expressed in heterologous systems (19). Second, flavonols have antioxidant capability *in vitro* (5, 20) and mutants that have impaired flavonol biosynthesis have higher levels of ROS in guard cells, root hairs, and pollen tubes (9, 10, 14, 15). These two mechanisms may also be interconnected as auxin transport has been shown to be regulated by levels of ROS (21).

ROS can function as signaling molecules to regulate plant development, environmental responses, and hormone signaling (1, 22, 23). The low baseline levels of ROS allow local increases in ROS to act as important developmental signals. In *Arabidopsis*, ROS signaling has been implicated in stomatal closure (10, 14, 24, 25), pollen tube growth and development (15, 26, 27, 28), root hair elongation (9, 29), root gravitropism and phototropism (30, 31, 32), primary root elongation (33), the transition from cell proliferation to elongation in the root tip (34), and lateral root emergence (35, 36, 37). ROS signaling is also involved in the production of the casparian strip (38), and resistance to stress including hypoxia, salt stress, and ozone (39, 40, 41). Yet, ROS are also produced as a result of metabolism and stress (42, 43). Therefore, plant cells have elaborate mechanisms to keep ROS at low levels to prevent oxidative damage including the production of antioxidant enzymes (42, 44, 45) and specialized metabolites, such as flavonols (1).

Flavonoids have been implicated in controlling root development (9, 18, 19, 46), yet whether specific flavonols control distinct aspects of root development has not been fully tested. The primary root forms within the embryo and emerges from the seedling. Lateral roots form post embryonically to increase root branching for stability and to maximize nutrient and water uptake (47, 48). The primary and lateral roots are then covered with small single cell projections called root hairs that maximize the surface area of roots (49). Lateral roots initiate from founder cells in the pericycle that remained competent to divide during their transition from the root apical meristem to the differentiation zone of the root (50). After founder cells begin to divide asymmetrically, the lateral root primordium (LRP) develops through a series of eight stages (50, 51, 52) where the LRP expands through the endodermis, cortex, and finally emerges through the epidermis. This process is regulated by mechanical signals (53, 54, 55, 56), hormonal signals including auxin (53), and biochemical signals such as ROS or nitric oxide (57). Flavonoids have been suggested to regulate lateral root development, based on an increased number of lateral roots in plants with mutations in the gene encoding the first committed step of flavonoid biosynthesis catalyzed by chalcone synthase. Although both *tt4(2YY6)* (18) and *tt4-1* (58) mutants form more lateral roots than wild-type, the specific flavonoids that modulate this process have not been examined, and it is not clear whether this function is through flavonoid antioxidant activity. This contrasts with root hairs, whose synthesis is also repressed by flavonols, with a specific role for one flavonol, quercetin in controlling root hair formation *via* modulating ROS levels (9).

This study examined the role of flavonols in modulating lateral root development and tested the hypothesis that flavonoids regulate this developmental process by scavenging ROS within the LRP. We quantified primordia and emerged lateral roots in a suite of flavonoid biosynthetic mutants, revealing that mutants that produce no flavonols had increased lateral root emergence. In contrast, a mutant that produces kaempferol at higher than wild-type levels and produces no other flavonols had fewer emerged lateral roots than wild-type, suggesting that kaempferol or downstream derivatives of this flavonol negatively regulate lateral root emergence. Consistent with an inhibitory effect of kaempferol, there is a negative correlation between the amount of kaempferol and the number of emerged lateral roots across multiple mutant lines. Using confocal microscopy and dyes that fluoresce upon binding flavonols or in response to oxidation by ROS, we find that in regions of roots with high levels of flavonols, there is decreased ROS abundance and in areas of high ROS, there are low concentrations of flavonols. These experiments support the model that flavonol-modulated root development is orchestrated by the scavenging of ROS within the lateral root primordia.

## Results

### Flavonoid mutants have predicted metabolite profiles

**Figure 1:**
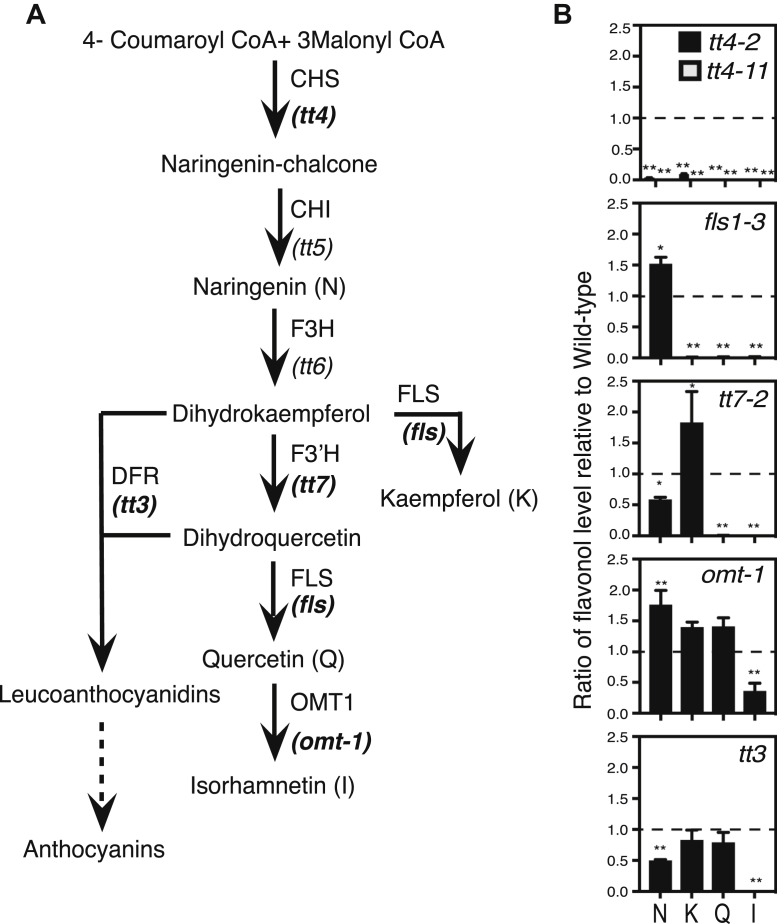
**Flavonoid biosynthesis mutants have expected flavonol profiles.***A*, The flavonoid biosynthesis pathway in *Arabidopsis* has mutants at each step indicated in italics under the enzyme. Mutants in *bold* were used in the experiments in this manuscript. *B*, Flavonol levels were determined by LC-MS and the amount in nmole/gfw (gram fresh weight) was calculated. The ratio of the metabolite concentration in the mutant was divided by the wild-type levels reported in the mutants. A *dashed line* indicates a ratio of 1, which is when metabolites are equivalent to wild-type levels. Statistics were determined using a one-way ANOVA on the ratio values followed by a Dunnett's post-hoc test. Significance is indicated by ∗*p* < 0.05, ∗∗*p* < 0.005. Data was collected from three separate experiments with total n of Col-0 (n = 22), *fls1-3* (n = 4), *tt4-2* (n = 12), *tt4-11* (n = 6), *tt7-2* (n = 5), *omt-1* (n = 7), and *tt3* (n = 11).

The flavonoid biosynthetic pathway is well characterized in *Arabidopsis*, with mutations mapped to the genes encoding each biosynthetic enzyme. The flavonoid biosynthetic pathway and the enzymes defective in these mutants are shown in Figure 1. We verified the metabolite profiles in the roots of the mutant alleles used in this study and under our specific growth conditions using liquid chromatography–mass spectroscopy (LC-MS). Flavonols underwent acid hydrolysis to remove any glycosylation prior to MS1 analysis to determine their concentration with standard curves and the aglycone flavonols were verified by MS2 (Table S1). Flavonol levels were quantified in Col-0 and six mutants with defects in genes encoding enzymes involved in flavonoid biosynthesis. The flavonol abundance relative to Col-0 for each mutant is shown in Figure 1 and the absolute value of each flavonol in nanomole per gram fresh weight (nmol/gfw) is reported in Table 1. In Col-0, naringenin and isorhamnetin were found at low concentrations, suggesting an efficient conversion of naringenin, an early pathway intermediate, into flavonols, and limited conversion of quercetin into isorhamnetin (Table 1).

**Table 1:** Quantification of flavonoids in roots of 7-day-old seedlings by liquid chromatography mass spectrometry reported in nmole/gfw

Genotype	Naringenin	Kaempferol	Quercetin	Isorhamnetin	Total
Col-0 (WT)	0.39 ± 0.08	200.2 ± 22.1	354.6 ± 42.5	18.44 ± 6.1	573.7 ± 67.1
*tt4-2*	0.01 ±0.003∗	16.3 ± 3.9∗	0.40 ± 0.09∗	N.D.	16.6 ± 4.0∗
*tt4-11*	N.D.	N.D.	0.01 ± 0.01∗	N.D.	0.01 ± 0.01∗
*tt7-2*	0.22 ± 0.02∗	363.3 ± 115.6∗	2.60 ± 0.8∗	N.D.	366 ±129∗
*fls1-3*	0.58 ± 0.05∗	1.8 ± 0.8∗	5.03 ± 1.6∗	0.27 ± 0.10∗	7.8 ± 2.4∗
*omt-1*	0.67 ± 0.10∗	276.9 ± 19.5∗	493.7 ± 55.5∗	6.3 ± 4.3∗	777.6 ± 73.2∗
*tt3*	0.19 ± 0.01∗	162.3 ± 36.2∗	274.5 ± 63.7∗	0.01 ± 0.003∗	436.9 ± 99.4∗

Statistics were measured by a one-way ANOVA with a Dunnett's post-hoc test over three separate replicates with an n = 4–21 (∗*p* < 0.0001). N.D. indicates values that were not detected because they were below the lowest concentration on the standard curve.

The flavonol accumulation profiles in the mutants are largely consistent with mutant enzyme function in the biochemical pathway (Fig. 1). The *tt4-2* and *tt4-11* mutants have defects in the gene encoding CHS, while the *fls1-3* mutant has a defect in the gene encoding flavonol synthase1 (FLS1), which is the predominant FLS isozyme catalyzing the final step of flavonol biosynthesis (59, 60). The *tt4-2*, *tt4-11*, and *fls1-3* mutants have background levels of the three flavonols: kaempferol, quercetin, and isorhamnetin. The *fls1*-3 mutant has significantly increased levels of naringenin, likely due to limited conversion to downstream products, while the *tt4* mutants have background levels of naringenin. The *tt7-2* mutant has a defect in the gene encoding the branch point enzyme Flavonoid 3′-Hydroxylase (F3′H). It produces no quercetin but has 1.8-fold higher levels of kaempferol. The *omt-1* mutant, which cannot convert quercetin to isorhamnetin, accumulates threefold lower levels of isorhamnetin than Col-0. The levels of kaempferol and quercetin in *omt-1* are significantly increased by 1.4-fold compared with Col-0 likely due to low flux toward isorhamnetin in this mutant. Interestingly, there is still production of isorhamnetin in *omt-1*, as mutants in the gene encoding this enzyme have been reported to have residual activity to act on the quercetin substrate (61) suggesting that another enzyme may also catalyze this reaction. The *tt3* mutant has a defect in the gene encoding dihydroflavonol 4-reductase (DFR), which converts dihydroxyflavonols to anthocyanins. There is an absence of DFR transcript and protein in roots of Col-0 (11, 46). This mutant therefore is not expected to have large differences in metabolites in roots that don't make anthocyanins. Interestingly, there are 10–20% reductions in levels of the three flavonols in *tt3*, which may indicate altered pathway regulation. The flavonol profiles of the most important mutants in the analyses below: *tt4*, *fls1*, and *tt7* have flavonoid profiles in roots consistent with their genetic defect.

### Mutants with impaired flavonol synthesis have increased lateral root number

**Figure 2:**
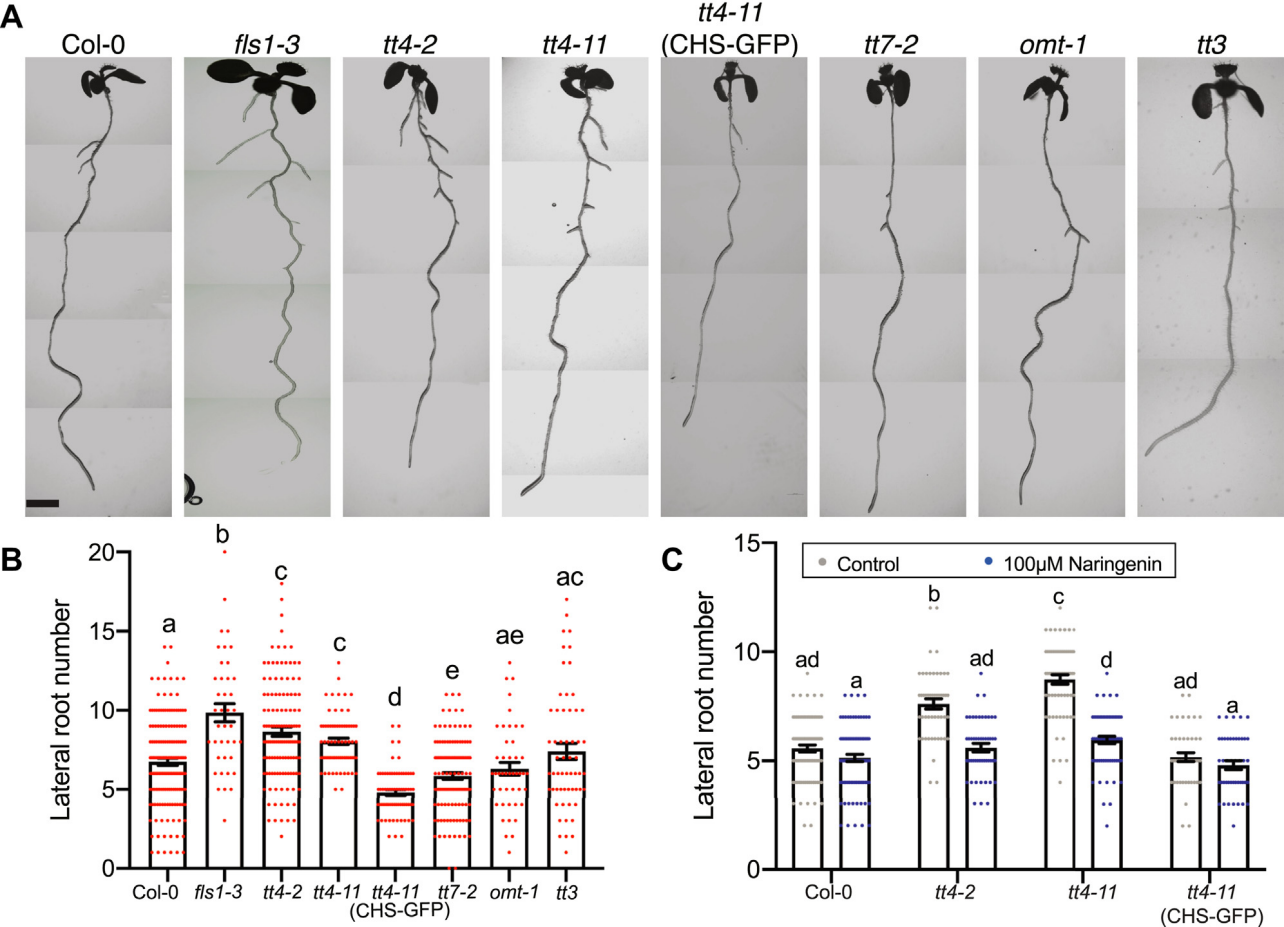
**Mutants in the flavonoid biosynthetic pathway suggest that kaempferol or a downstream derivative is negatively modulating lateral root emergence.***A*, Representative images of each mutant were taken using a Zeiss Axiozoom microscope. Scale bar: 2 mm. *B*, Lateral root number was quantified over four replicates with total number of seedlings: Col-0 (n = 161), *fls1-3* (n = 40), *tt4-2* (n = 122), *tt4-11* (n = 67), CHS-GFP (n = 69), *tt7-2* (n = 121), *omt-1* (n = 45), and *tt3* (n = 58). Statistics were performed using a one-way ANOVA with *p* < 0.05. *C*, Lateral root number was evaluated after a 3-day treatment with 100 μM naringenin. The average and SE are from four independent experiments with the following total n: Col-0 (EtOH: n = 89; Naringenin: n = 89), *tt4-2* (EtOH: n = 50; Naringenin: n = 50), *tt4-11* (EtOH: n = 59; Naringenin: n = 64), and CHS-GFP (EtOH: n = 45; Naringenin: n = 45). Statistics were measured using a two-way ANOVA followed by a Tukey post-hoc test *p*< 0.05. *Bars* with the same letter represent no statistical difference, while different letters indicate values that are significantly different.

To evaluate root development in the absence of all flavonols, lateral root number of 8-day-old seedlings was examined in Col-0 and three mutants that do not produce flavonols. Representative images of the lateral root phenotypes of *tt4*-*2*, *tt4*-*11*, and *fls1-3* are shown in Figure 2*A* and the number of emerged lateral roots in 8-day-old seedlings is shown in Figure 2*B*. There are significantly more lateral roots in both *tt4* alleles as compared with the wild-type, consistent with a prior report using the *tt4(2YY6)* allele (18). The *fls1-3* mutant, which synthesizes all the pathway intermediates but makes no flavonols, also has significantly more lateral roots than Col-0. This result suggests that the flavonols, rather than their precursors, limit the formation and/or emergence of lateral roots in these flavonol deficient mutants.

### Genetic and chemical complementation of *tt4-2* and *tt4-11* reduce lateral root number to wild-type levels

The *tt4-11* mutant was complemented both genetically and chemically to verify the loss of function of *CHS* resulted in the increased lateral root phenotype. The *tt4-11* mutant was genetically complemented with the *CHSpro::CHS-GFP* transgene (CHS-GFP) and an image of this genotype is shown in Figure 2*A,* while the lateral root number was quantified in Figure 2*B*. The genetic complementation of *tt4-11* with CHS-GFP resulted in significantly fewer lateral roots compared with the untransformed *tt4-11* mutant to levels lower than Col-0. The levels of flavonols in this complemented line were measured to ask if the decreased number of lateral roots were due to elevated flavonol content (Table S2). The abundance of kaempferol, quercetin, and isorhamnetin was at 75% of the values of Col-0, although these differences were not statistically significant.

We also chemically complemented *tt4-2* and *tt4-11* with the flavonol precursor naringenin, a pathway intermediate produced downstream of CHS. Five-day-old seedlings of Col-0, the two *tt4* alleles, and *tt4-11* (CHS-GFP) were transferred to media supplemented with 100 μM naringenin for 3 days before the number of lateral roots was quantified. The lateral root numbers in *tt4-2* and *tt4-11* were significantly reduced by naringenin treatment to wild-type levels. In Col-0 and the *tt4-11*(CHS-GFP) line, which produce naringenin, the number of lateral roots was not significantly different in the presence or absence of exogenous naringenin shown in Figure 2*C*. The absence of an effect with naringenin supplementation in Col-0 and the complemented line is consistent with the conversion of naringenin to dihydrokaempferol being rate limiting. These complementation results are consistent with impaired flavonol synthesis leading to an increased number of lateral roots.

### Flavonoid biosynthesis mutants reveal the pathway intermediates that regulate root development

Lateral root development was evaluated in mutant seedlings with defects in genes encoding enzymes at multiple steps in the flavonoid biosynthetic pathway to determine whether a specific flavonol functions to modulate this development. Representative images of these roots are shown in Figure 2*A* and the number of emerged lateral roots were quantified and are reported in a bar graph overlaid with individual data points in Figure 2*B*.

The lateral root number of the *omt-1* and *tt3* mutants were not significantly different from wild-type, consistent with the absence of root developmental roles of isorhamnetin, anthocyanins, and other downstream molecules in the flavonoid pathway. The mutant *tt7-2*, which has higher levels of kaempferol and for which quercetin and isorhamnetin are not synthesized, has a decreased number of lateral roots (Fig. 2*B*). Similarly, the *tt7-1* allele also had a decreased number of lateral roots compared with the Col-0 wild-type (Fig. S1). The root developmental pattern of *tt7-2* and *tt7-1* suggests either kaempferol or a downstream product inhibits lateral root formation/emergence or that local concentration changes of kaempferol regulate development.

To verify that the effects on lateral root number were not tied to difference in primary root formation or elongation, we quantified root length across these genotypes. There were no significant differences in length in *tt4*-*2*, *tt4*-*11*, *fls1*-*3*, *tt7*-*2*, and *tt3* mutants compared with the wild-type (Fig. S2) although *omt-1* has a ∼17% reduction on root length. Therefore, the effect of mutations that alter flavonol biosynthesis on lateral root development was independent of primary root length.

We used chemical complementation of flavonol-biosynthetic mutants to further evaluate the relationship between flavonols and lateral roots similar to our previous treatments with the flavonol precursor, naringenin. We also treated the *fls1-3* mutant with naringenin and find a smaller naringenin effect than in *tt4* alleles, consistent with the defect in flavonol biosynthesis in this mutant being downstream of naringenin synthesis in the flavonol biosynthetic pathway (Fig. S3*A*). A small decrease in lateral root number by naringenin treatment may be explained by another FLS enzyme converting naringenin to downstream flavonol metabolites, a point that is consistent with low levels of flavonols in the *fls1-3* mutant allele and with discussions in the literature (59, 60). To identify the active flavonol, we treated 5-day-old Col-0 and flavonol deficient seedlings (*tt4*-*2*, *tt4*-*11*, and *fls1-3*) with kaempferol or quercetin at 100 μM for 3 days (Fig. S3*B*). Flavonol compounds are more hydrophobic than naringenin, thus limiting their uptake into cells, which is why this higher dose was used. In Col-0 quercetin treatment surprisingly increased the number of emerged lateral roots. The *tt4-2* and *fls1-3* mutants had significantly decreased lateral root emergence after treatment with both kaempferol and quercetin, with *tt4-2* lateral root numbers reduced to wild-type levels. The ability of both flavonols to complement these mutants is consistent with the presence of enzymes that can interconvert these two metabolites. Together these results indicate that the lateral root number is inversely proportion to the presence of endogenous flavonols, with the levels of kaempferol being particularly important.

### Flavonol mutants show altered lateral root emergence

**Figure 3:**
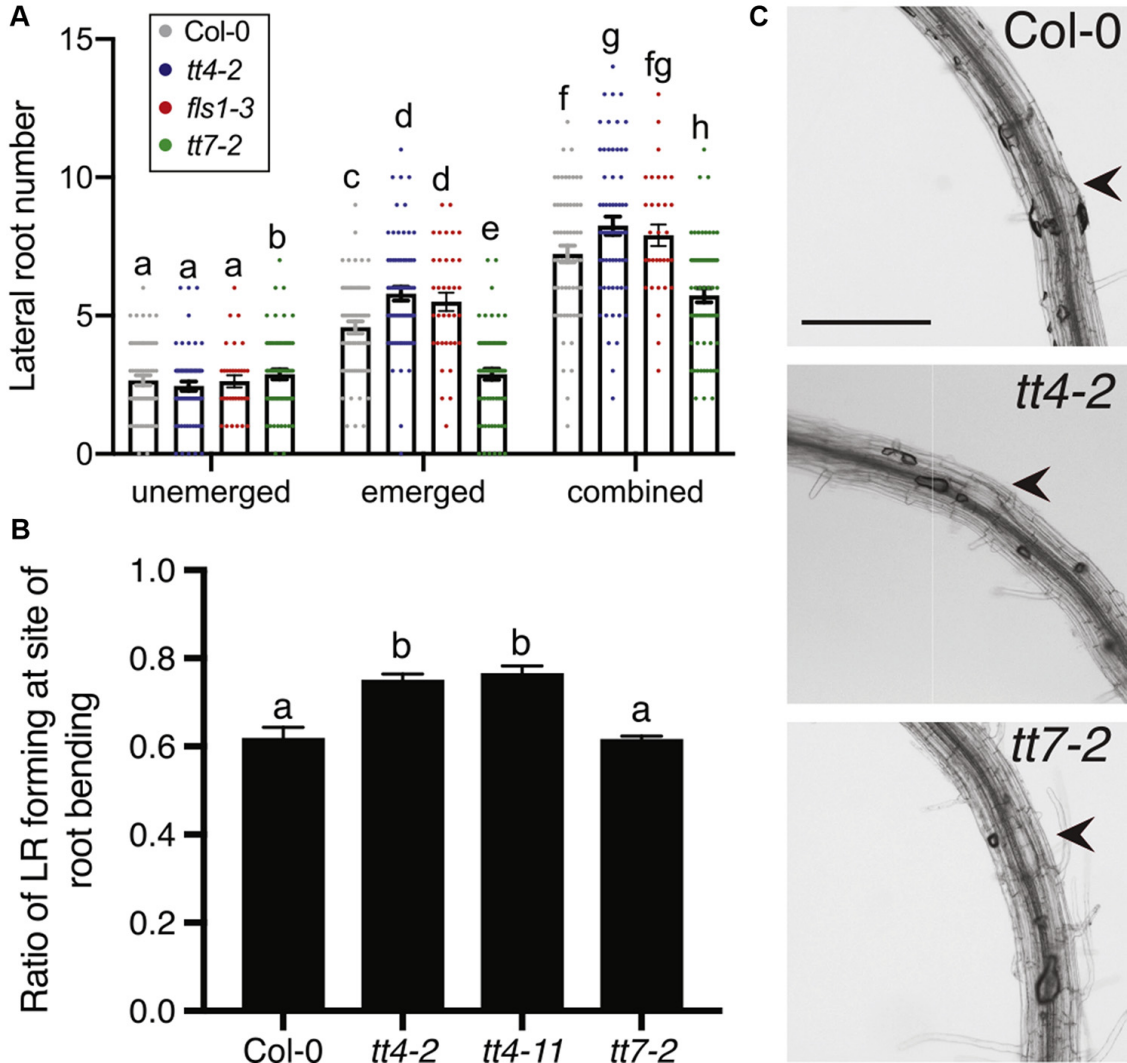
**Flavonol mutants have altered lateral root emergence.***A*, The number of lateral primordia (Stages 4–7) and emerged lateral roots, and the combined totals were quantified in cleared 7-day-old seedlings. The average and SE from four separate experiments with a total n = 60–68. Statistically significantly different values were determined using a one-way ANOVA with *p* < 0.05. *Bars* with the same letter indicate no statistical significance while bars with different letters are statistically significant from one another within each subgroup of unemerged (a–b), emerged (c–e), and combined (f–h). *B*, Roots were bent after 5 days of growth to a 90°-angle and determination of lateral root formation was done 2 days later. The average and SE from five separate experiments with a total n: (Col-0 n = 71; *tt4-2* n = 44; *tt4-11* n = 72; *tt7-2* n = 70) are reported. Statistical differences were determined using a one-way ANOVA with *p* < 0.0001 and indicated with different letters representing significant differences. *C*, representative images of lateral root primordia in bent roots indicated by *black arrows*. Scale bar = 50 μm.

We asked whether the increase in lateral root number in the *tt4* alleles and decrease in *tt7-2* were due to modulation of lateral root emergence, lateral root initiation, or a combination of both. Lateral root initiation begins when founder cells exhibit an asymmetric division (stage 1), which is then followed by a precise series of divisions to form LRP that ultimately move through the epidermis as emerged lateral roots (stage 8) (50). To determine the LR developmental stage at which lateral roots were impaired in *tt7*-*2*, we quantified the number of LRP between stage 3 and stage 8 and emerged lateral roots in Col-0 and *tt4*-*2*, *fls1*-*3*, and *tt7-2* (Fig. 3*A*) and in *tt4*-*11* and *omt*-*1* (Fig. S4). The number of LRP in *tt4*-*2*, *tt4*-*11*, and *fls1-3* mutants were not significantly different from Col-0. In contrast, these genotypes all had a significantly higher number of emerged lateral roots than Col-0. The *tt7-2* mutant had slightly but significantly increased LRP compared with Col-0, but had a larger and significant decrease in the number of emerged lateral roots relative to Col-0 (Fig. 3*A*). Additionally, the *tt4-11* mutant complemented with the *CHS-GFP* transgene, reversed the increased emerged lateral root phenotype for *tt4-11* to wild type levels, but there was no difference in unemerged lateral roots between these genotypes and wild-type. This result indicates that in *tt4*-*2*, *tt4*-*11*, and *fls1-3* mutants lateral root emergence is enhanced, while in *tt7-2* emergence is impaired.

In *Arabidopsis*, a 90-degree bend in the primary root leads to the production of a lateral root at the position of the bend (54, 55, 56). We took 5-day-old seedlings and reoriented them with a 90-degree angle at a 2 mm distance from the root tip. The formation of an LRP or emerged LR was evaluated after 48 h with representative images shown in Figure 3*C*. The number of roots with lateral roots (emerged or unemerged) at the bend position was divided by the total number of roots to derive the ratio reported in Figure 3*B*. The ratios calculated in mutants were compared with wild-type. Col-0 and *tt7-2* formed a lateral root in 61% of roots that were bent. The two *tt4* mutant alleles, *tt4-2* and *tt4*-*11*, formed significantly more lateral roots than wild-type, with 75 and 76% of the bent roots initiating lateral roots, respectively. The increase in lateral root initiation in the *tt4* mutants likely contributes to the increased lateral root phenotype. These data indicate that the decrease in lateral root number in *tt7-2* is likely due to decreased emergence, not a decrease in lateral root initiation.

### Kaempferol level negatively correlates with lateral root number

**Figure 4:**
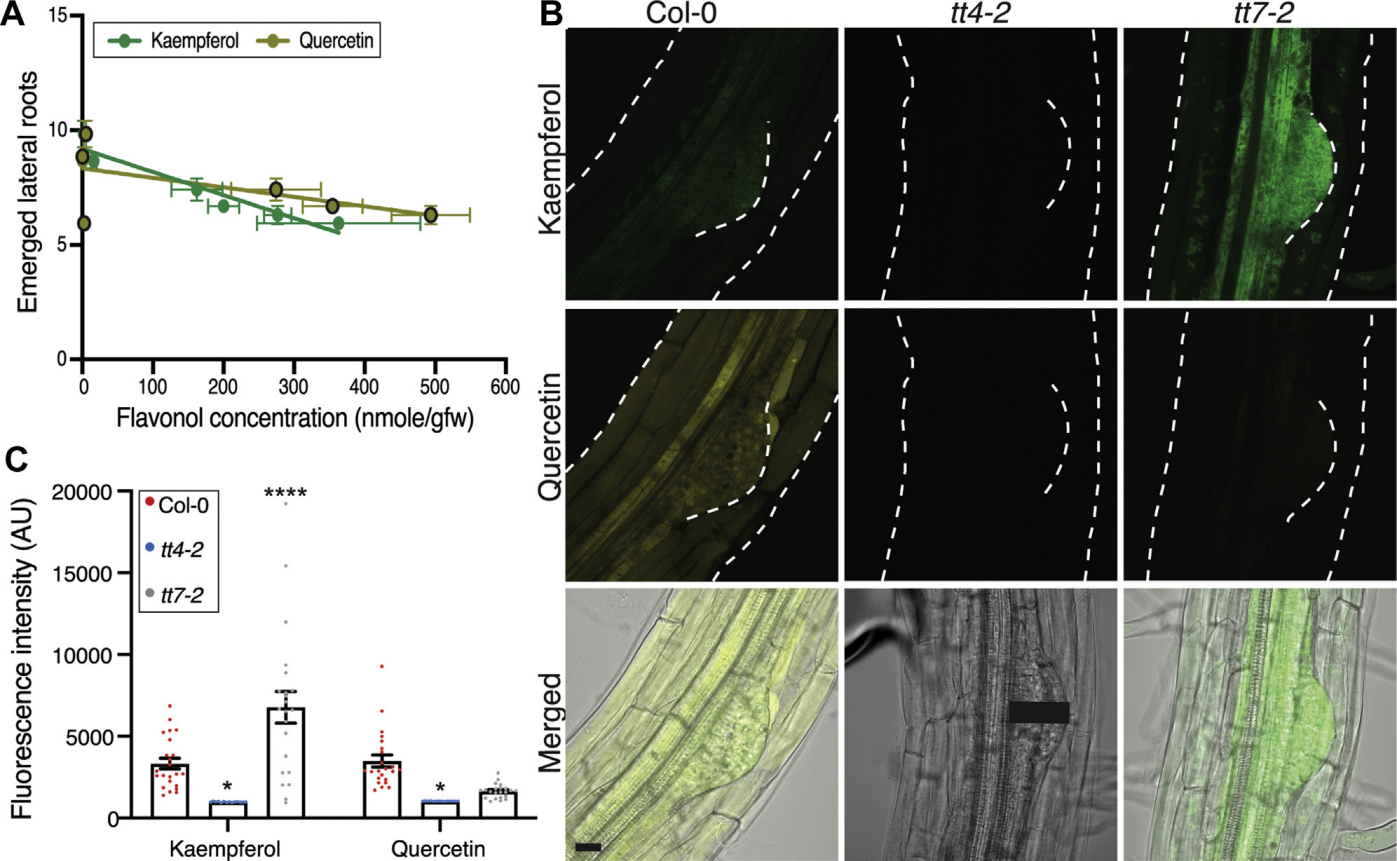
**Kaempferol accumulates in lateral root primordia**. *A*, The number of emerged lateral roots was plotted as a function of concentration of kaempferol or quercetin for wild-type and the suite of flavonol mutants. Results from a linear regression are drawn and the *p* value and R square are: kaempferol (*p* = 0.0032, R^2^ = 0.9094) and Quercetin (*p* = 0.1644, R^2^ = 0.3465). Kaempferol has a significantly negative correlation with emerged lateral root number. *B*, Representative images of 8-day-old seedlings stained with DPBA are shown with K-DPBA (*green*) and Q-DPBA (*yellow*) imaged using two channel mode. Scale bar: 50 μm. *C*, DPBA fluorescence intensity was quantified for K-DPBA and Q-DPBA using a 100-pixel line (11.5 μm wide) drawn from the vasculature to the tip of the primordia, as represented by the *black line* on the *tt4-2* DIC image. The average fluorescence of multiple roots taken at identical gain, laser intensity, and pinhole settings are shown. Statistics were calculated based on a two-way ANOVA with a Tukey post-hoc test with a total n: Col-0 (n = 23), *tt4-2* (n = 13), *tt7-2* (n = 22). Mutant values were compared with wild-type (∗*p* < 0.05, ∗∗∗∗*p* < 0.0001).

To understand why *tt7-2* has an opposite lateral root phenotype compared with the flavonol-deficient mutants, we asked whether the number of emerged lateral roots was predicted by the amount of either quercetin, kaempferol, or the total flavonol level. We plotted the average number of emerged lateral roots in wild-type and mutants as a function of either the quercetin or kaempferol concentration (Fig. 4*A*) followed by a linear regression analysis. While there was no significant correlation between the number of emerged lateral roots and the levels of quercetin, there was a significant negative correlation between the number of lateral roots and the level of kaempferol. These data suggest that the concentration of kaempferol, or its downstream metabolites, is pertinent to lateral root emergence, leading us to ask whether the site of accumulation of kaempferol or just the amount of this flavonol control lateral root formation.

### Flavonols accumulate within and around developing lateral root primordia

To understand how flavonols regulate lateral root development, we asked where kaempferol and quercetin accumulate in LRP and emerging lateral roots. We stained roots of 8-day-old *Arabidopsis* seedlings with diphenylboric acid 2-aminoethyl ester (DPBA), a dye that becomes fluorescent upon binding kaempferol and quercetin (11, 62). The emission spectra resulting from DPBA-bound kaempferol (K-DPBA) can be resolved from DPBA-bound quercetin (Q-DPBA), and these two fluorescent signals can be quantified by laser scanning confocal microscopy (LSCM) (11).

Confocal images of DPBA-stained Col-0 with K-DPBA shown in green and Q-DPBA shown in yellow illustrate the accumulation of these two compounds (Fig. 4*B*). In Col-0, both K- and Q-DPBA fluorescent signals are found in pericycle cells that give rise to LRP and within LRP, as well as in surrounding tissues (Fig. 4*B*). To verify the specificity of DPBA for recognition of flavonols in mature regions of the root, we examined the *tt4-2* mutant, which produces neither flavonol. Consistent with expectations, *tt4-2* had only background levels of K- and Q-DPBA fluorescence (Fig. 4*B*).

As two *tt7* mutant alleles had reduced root formation and *tt7-2* had higher concentration of kaempferol and its glycosides, we wanted to understand whether there was cell-type-specific accumulation of flavonols in this genotype. In the *tt7-2* mutant, K-DPBA fluorescence is evident in Figure 4*B*, with Q-DPBA signal at background levels consistent with the flavonol profile in Figure 1. In *tt7-2*, there is a striking increase in levels of K-DPBA fluorescence in primordia. To determine whether the K-DPBA signal is consistently higher within LRP of *tt7-2* across multiple samples, the average fluorescence was quantified using a line drawn from the vasculature to the edge of the primordium through the middle of the LRP, as illustrated with the black line in Figure 4*B*. The vasculature is evident in differential interference contrast (DIC) images as the tissue directly below the pericycle. A line width of 11.5 μM was used to account for any variance between flavonol accumulations that may occur across the width of the LRP. The K-DPBA and Q-DPBA fluorescence is found within primordia of Col-0, and both signals are at background levels in *tt4-2*. In *tt7-2*, the K-DPBA signal in lateral root primordia is significantly higher (two-fold elevated) over levels in Col-0 (*p* < 0.0001), while Q-DPBA fluorescence is at background levels and is not statistically different from those of *tt4-2*, consistent with the absence of quercetin in both mutants (Fig. 4*B*). The two-fold increase in K-DPBA signal in primordia is similar to the two-fold increase of kaempferol concentration detected by LC-MS (Fig. 1). This pattern suggests that kaempferol accumulation in lateral root primordia is appropriately positioned to modulate lateral root development.

### Flavonol biosynthetic enzymes reporter fusions are expressed in pericycle cells and emerged lateral roots

**Figure 5:**
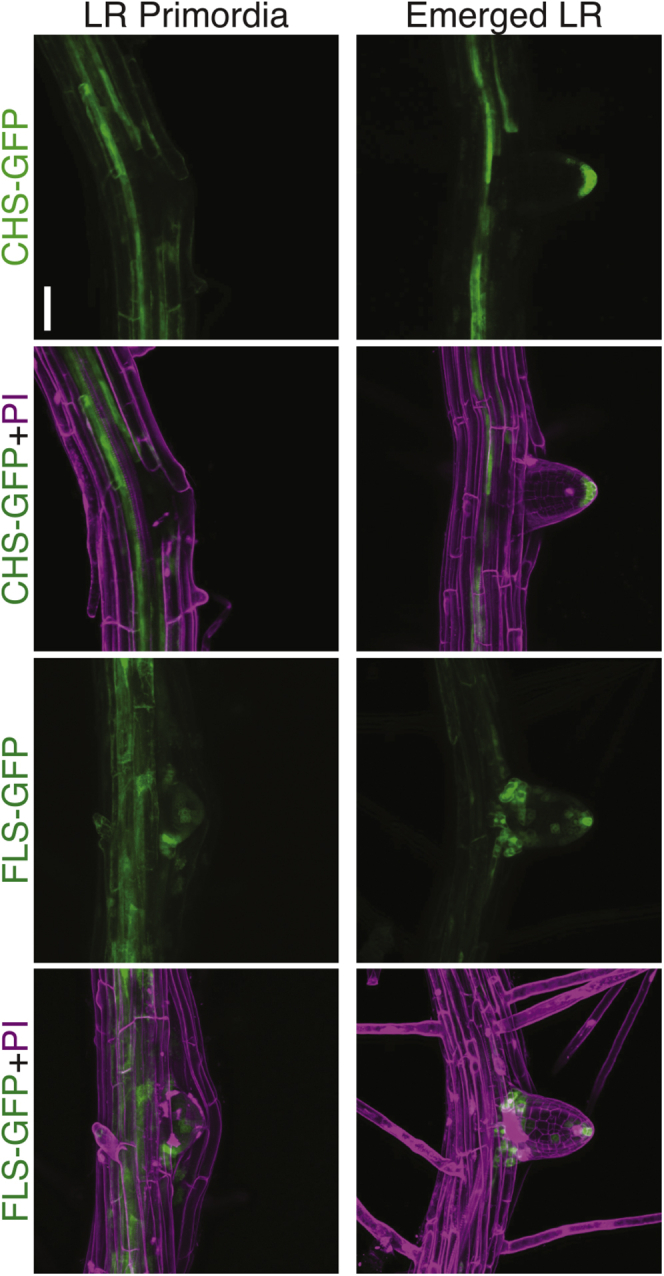
***CHSpro:CHS-GFP* and *FLSpro:FLS-GFP* accumulate in the emerged lateral root.** CHS-GFP and FLS-GFP 8-day-old seedlings were stained with propidium iodide shown in magenta. PI detects demethoxylated pectin in the cell wall, revealing the cellular structure of the root. Representative images are from three separate experiments with identical gain, laser intensity, and pinhole. Scale bar: 50 μm.

Flavonols accumulated in founder cells and LRP, leading us to ask where their biosynthetic machinery localizes. We used two fluorescent protein constructs, *CHSpro:CHS:GFP* (CHS-GFP) and *FLS1pro:FLS1:GFP* (FLS-GFP) (11, 13), to visualize by LSCM where these key enzymes accumulate during lateral root development. Figure 5 shows maximum intensity projections of representative images of LRP and emerged lateral roots expressing CHS-GFP and FLS-GFP and stained with propidium iodide, which highlights the cell wall by binding to demothoxylated pectin (63). GFP fluorescence of both reporters was detected in the pericycle cell files that give rise to lateral roots and at the base and the apical tip of emerged lateral roots (Fig. 5). CHS-GFP signal was not detected in the LRP, while FLS-GFP signal at the tip of the LRP parallels the signal at the tip of emerged lateral roots. These data are consistent with flavonol synthesis in pericycle cells prior to formation of LRP or with a precursor for flavonols being transported to primordia for the production of flavonols. Prior reports indicate that naringenin can move long distances in roots, consistent with this later possibility (64, 65).

### Antioxidant treatment reduces lateral root number and ROS levels in *tt4*

**Figure 6:**
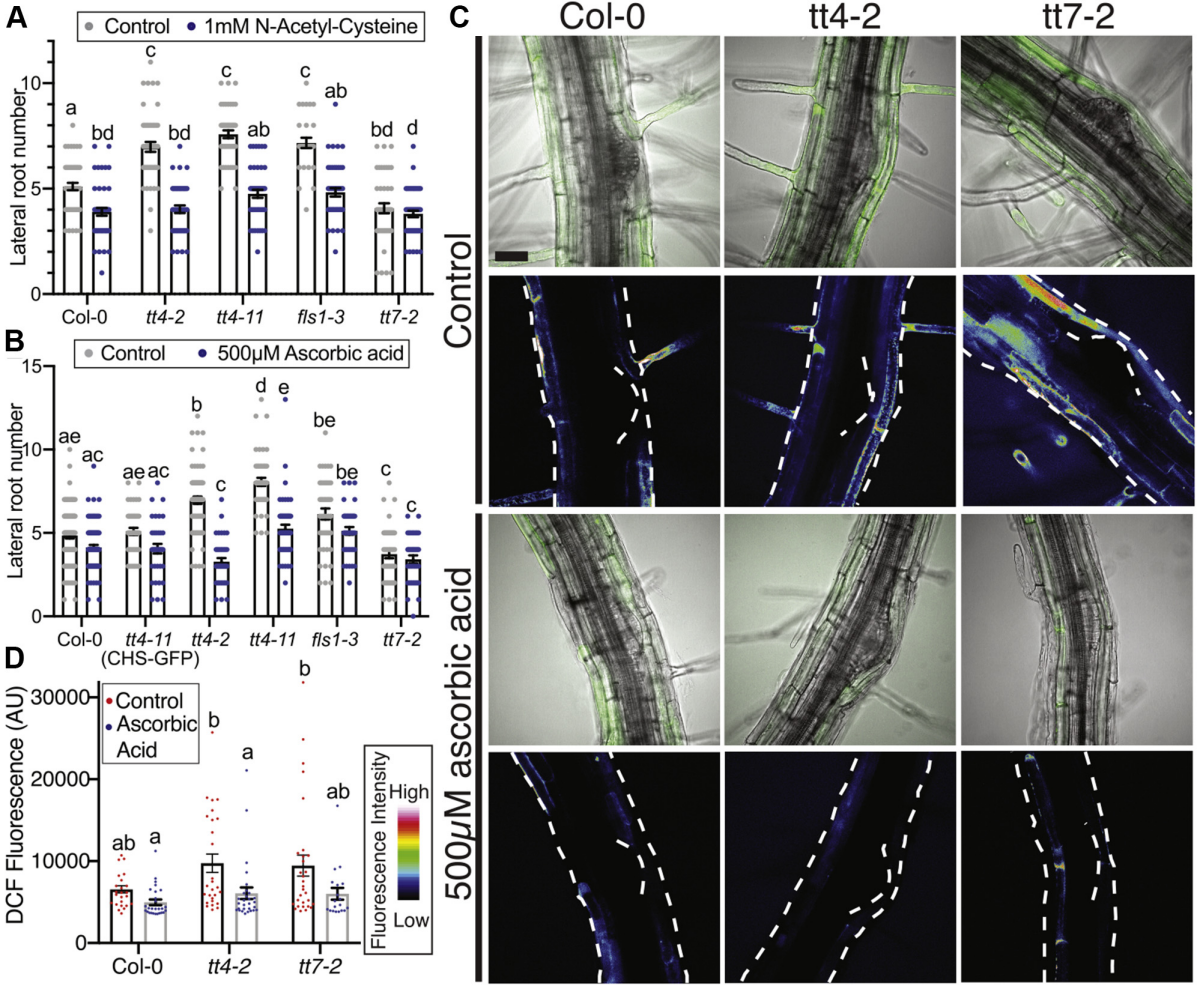
**Treatment with antioxidants reduces lateral root number and DCF signal in *tt4-2*.***A*, Lateral root number was evaluated after a 3-day treatment with 1 mM N-acetyl-cysteine. Data is summarized from three independent experiments with an n of: Col-0 (control: n = 50; NAC: n = 50), *tt4-2* (control: n = 49; NAC: n = 45), *tt4-11* (control: n = 45; NAC: n = 45), *fls1-3* (control: n = 30; NAC: n = 45), *tt7-2* (control: n = 45; NAC: n = 50). *B*, Lateral root number was evaluated after a 3-day treatment with 500 μM ascorbic acid. Data summarized from four independent experiments with varying total numbers: Col-0 (control: n = 93; ascorbic acid: n = 89), *tt4-11*(CHS-GFP) (control: n = 43; ascorbic acid: n = 36), *tt4-2* (control: n = 74; ascorbic acid: n = 39), *tt4-11* (control: n = 48; ascorbic acid: n = 55), *fls1-2* (control: n = 44; ascorbic acid: n = 40) and *tt7-2* (control: n = 44; ascorbic acid: n = 38). *C*, Eight-day-old seedlings were stained with DCF and immediately imaged at the same laser power, gain, and pinhole for three separate experiments. DCF is oxidized by most ROS, so is a general ROS sensor. The DCF signal is reported directly as an overlay to the DIC and is also shown as a heatmap using the Zen LUT feature. Lateral root primordia were stage 4 and older due to limited stain uptake in primordia younger than stage 4. Scale bar: 50 μm. *D*, DCF quantification data is summarized with a total n: Col-0 (control: n = 13; ascorbic acid: n = 19), *tt4-2* (control: n = 15; ascorbic acid n = 21), *tt7-2* (control: n = 18; ascorbic acid: n = 17). Statistics were measured using a two-way ANOVA with a post-hoc Tukey test. *Bars* with the same letter represent no statistical difference, while different letters indicate statistical significance with a *p* < 0.05.

We asked if the mechanism by which flavonol-deficient mutants have elevated lateral roots is through increases in ROS signaling, due to the absence of flavonol antioxidants. To test this hypothesis, we used two ROS scavengers, ascorbic acid, which has been shown to scavenge radical species (66), and N-acetyl-cysteine (NAC), which reduces levels of multiple ROS, but especially peroxides (67, 68). We asked if the enhanced lateral root phenotype in the *tt4* and *fls1-3* mutants was reversed by antioxidant treatment. Seedlings were grown on MS media for 5 days and then transferred to media containing 500 μM ascorbic acid or 1 mM NAC for 3 days before lateral root number was quantified. Treatment with NAC significantly reduced the lateral root number in Col-0, the *tt4* mutants, and *fls1*-*3*, but had no effect on *tt7-2* (Fig. 6*A*). Treatment with ascorbic acid significantly reduced the lateral root number in both *tt4* mutant alleles to levels similar to or less than wild-type, consistent with elevated ROS driving the increased lateral root emergence (Fig. 6*B*). In contrast, the ascorbic acid treatment had no significant effect on Col-0 and neither antioxidant altered lateral root development in *tt7*-*2*. Together, these results suggest that two antioxidants can reverse the increases in lateral roots in flavonol defient mutants and that synthesis of a kaempferol antioxidant is sufficient to eliminate the effect of exogenous antioxidant treatment.

### Fluorescence of a general ROS sensor is elevated in flavonol mutants and reduced by antioxidants

To highlight where ROS accumulate across developing lateral roots and to examine the effect of ascorbic acid treatment on ROS levels, we examined levels of ROS in flavonol-deficient mutants in the absence and presence of ascorbic acid. We used a general ROS sensor, 2′,7′-dichlorodihydro-fluorescein diacetate (CM H_2_DCF-DA) and LSCM to visualize ROS in and around LRP. CM H_2_DCF-DA enters the cell through the plasma membrane, where the diacetate group is cleaved by intracellular esterases. The released DCF will fluoresce upon oxidation by ROS. DCF has a transient signal, which leads to fluorescence increases upon oxidation, but it can then be reduced and lose its fluorescence (69). DCF fluorescence is shown overlaid on the DIC image and is also shown as a heat map using the lookup table (LUT) module in Zeiss Zen software (Fig. 6*C*). DCF fluorescence is the highest in epidermal and cortical tissues above the lateral root primordia in wild-type. We quantified DCF signal in untreated roots using a region of interest over the LRP. The *tt4-2* and *tt7-2* mutants both had significantly higher DCF signal above LRP than wild-type (Fig. 6, *C*–*D*). Ascorbic acid treatment of Col-0 led to a reduction of DCF signal over the lateral root primordia as compared with untreated samples. Similarly, treatment of *tt4-2* and *tt7-2* with ascorbic acid significantly reduced DCF signal in this region as compared with these genotypes without treatment. This reduction in DCF signals was to levels not significantly different from untreated Col-0, consistent with ascorbic acid acting as an antioxidant.

DCF signal was not observed inside the lateral root primordia, where we see high levels of flavonols. To determine whether the absence of DCF signal in the primordia is due to the inability of dye to be taken up in the primordia, we used the dye fluorescein diacetate (FDA) as a control, because it has a similar structure to DCF, but is not redox-sensitive. LRP of stage 4 and later were able to take up FDA (Fig. S5) even though they showed no DCF signal. Lateral root primordia of stage 1–3 had neither FDA nor DCF signal, presumably because dye uptake is limited at these earlier developmental stages. The low level of DCF signal in primordia of wild-type and flavonol mutants, even though FDA signal was detected, suggests ROS is present at low levels in the LRP. Although DCF is likely taken up in primordia, the transient nature of the signal diminishes our ability to evaluate ROS levels within the LRP.

### The *tt7-2* mutant has deceased superoxide (DHE) signal in the primordia compared with wild-type

**Figure 7:**
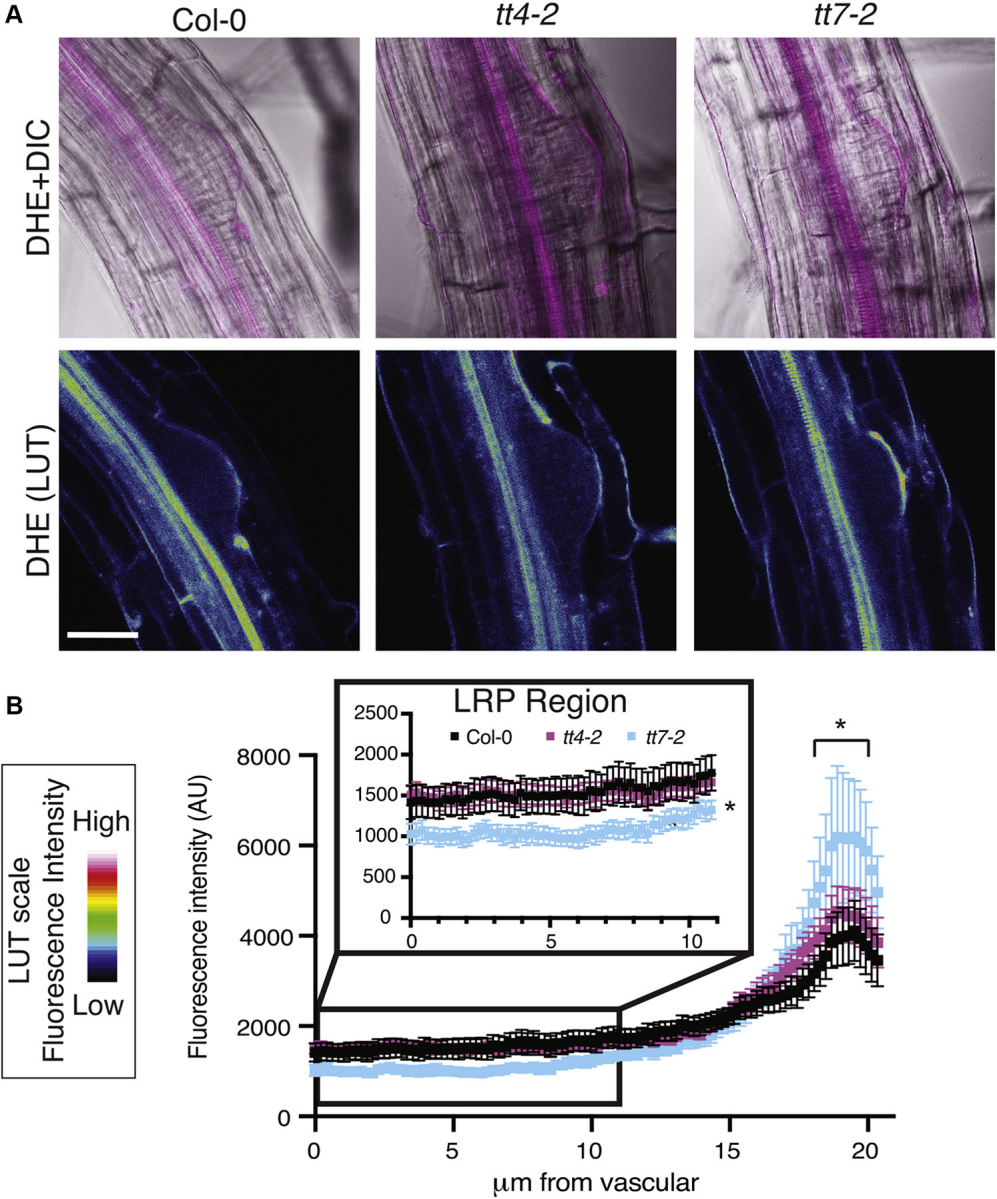
**The *tt7-2* mutant has decreased DHE signal within the lateral root primordia.***A*, Eight-day-old seedlings were stained with DHE and immediately imaged using identical imaging parameters over three replicates. DHE has selectivity for superoxide compounds. The DHE signal is visualized as an overlay on the DIC image and as a heat map generated using the LUT feature in Zen. *B*, The DHE signal was quantified using FIJI and an 11.5 μm was drawn from the base of the primordia to the tip of the primordia and the average and SE for 19–22 roots are reported. The *inset* within this panel focuses on the fluorescence intensity in the first 10 μm. Statistics were determined using a one-way ANOVA based on the average of each line (∗*p* < 0.05). Scale bar: 50 μm.

We used a superoxide-selective sensor, dihydroethidium (DHE), to monitor the accumulation of superoxide in wild-type, *tt4*-*2*, and *tt7-2*. DHE signal is generated by the oxidation of ethidium to ethidium^+^ (70). The DHE signal is reported two ways, with the signal overlaid on DIC, and as a heat map generated using the LUT feature in Zen (Fig. 7*A*). In all three genotypes, the DHE signal was at high levels in the vasculature and was visible within the LRP, with a brighter signal directly above the LRP. The different localization of these ROS sensors, DCF and DHE, may be due to the reversible oxidation and signal of DCF leading to a transient signal (69) or to the selectivity of DHE for superoxide (71). We quantified DHE fluorescence in the primordia with a region of interest drawn from the vasculature to the region directly above the primordia, and the fluorescence intensity was plotted as a function of the distance from the vasculature (Fig. 7*B*). The signal directly above the LRP is significantly higher in the *tt7-2* mutant, mirroring the increased signal above the LRP observed using DCF, although *tt4-2* is not significantly different from Col-0. The signal throughout the LRP in *tt4-2* is comparable with wild-type, while the signal in *tt7*-2 is significantly reduced, consistent with the presence of higher levels of kaempferol in *tt7-2* LRP. Therefore, we propose that the decreased levels of superoxide detected by DHE within the primordia of *tt7-2* lead to impaired lateral root emergence.

### Treatment with IAA increases lateral root number for all flavonoid mutants

**Figure 8:**
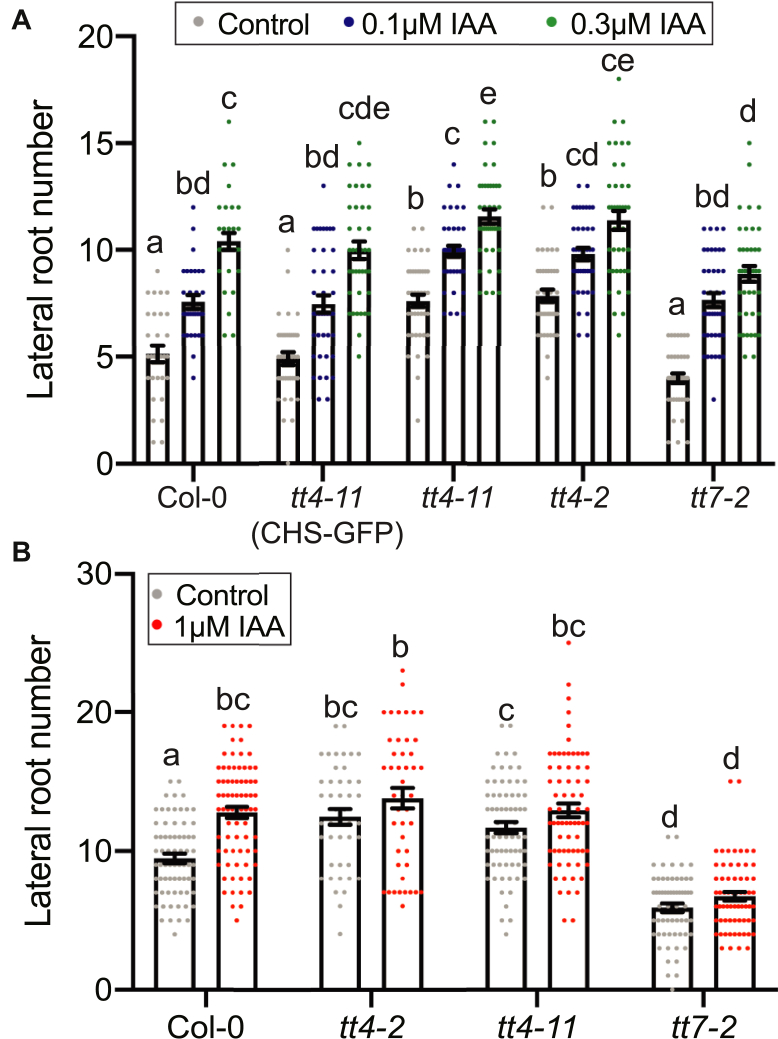
**Flavonol mutants show maximal auxin delivery to the root.***A*, Lateral root number was quantified for 8-day-old seedlings after a 3-day IAA treatment across three individual experiments for a total n of: Col-0 (control: n = 30; 0.1 μM IAA: n = 30; 0.3 μM IAA: n = 30), *tt4-11*(CHS-GFP) (control: n = 39; 0.1 μM IAA: n = 40; 0.3 μM IAA: n = 39), *tt4-2* (control: n = 39; 0.1 μM IAA: n = 40; 0.3 μM IAA: n = 40), *tt4-11* (control: n = 40; 0.1 μM IAA: n = 40; 0.3 μM IAA: n = 40), and *tt7-2* (control: n = 40; 0.1 μM IAA: n = 40; 0.3 μM IAA: n = 40). *B*, IAA-containing agar droplets were placed at the root–shoot junction of 5-day-old seedlings. Lateral root number was quantified for 9-day-old seedlings across six individual experiments for a total n of: Col-0 (control: n = 69; IAA: n = 75), *tt4-2* (control: n = 44; IAA: n = 45), *tt4-11* (control: n = 69; IAA: n = 70), and *tt7-2* (control: n = 60; IAA: n = 65). Statistics were performed using a two-way ANOVA with a Tukey post hoc test. Bars with the same letter represent no statistical difference, while different letters indicate values that are significantly different with a *p* < 0.05.

Since auxin drives initiation of LRP in pericycle cells and mutants with impaired flavonol synthesis have increased auxin transport (11, 17, 18), we tested an alternative hypothesis that increased LRP formation in flavonol mutants was due to increased auxin delivery to pericycle cells. To examine whether the effect of flavonols on root development was through altered auxin transport, we transferred 5-day-old seedlings of *tt4*-*2*, *tt4*-*11*, *tt4*-*11*(*CHS*-*GFP*), and *tt7-2* mutants to media containing 0.1 μM or 0.3 μM indole-3-acetic acid (IAA) for 3 days. Comparison of the number of lateral roots revealed that all mutants and the wild-type had increased number of emerged lateral roots after global treatment with IAA (Fig. 8*A*), resulting in equivalent number of LRs in all genotypes. These data could be interpreted as the *tt4* mutants being less sensitive to IAA or having maximal auxin delivery to sites of lateral root initiation. Overall, this data is not consistent with differences in auxin transport or signaling controlling the differences in lateral roots between flavonol biosynthetic mutants.

To further test the effect of auxin on lateral root emergence in the flavonol mutants, we used a localized IAA treatment at the root shoot junction and measured the number of emerged lateral roots. Agar droplets containing either 1 μM IAA in EtOH or EtOH were placed at the root–shoot junction of 5-day-old seedlings and lateral root number was counted after 4 days of treatment. A higher dose of IAA was used in this treatment, since the contact with the root was restricted to a narrow region, and a previous study demonstrated that a higher dose of IAA was needed to get a response to localized treatment (71). This treatment significantly increased lateral root number in Col-0 (Fig. 8*B*), consistent with rootward transport of auxin stimulating lateral root emergence (72, 73). In contrast to wild-type, the number of lateral roots was not significantly different in the presence or absence of IAA in *tt4*-*2*, *tt4*-*11*, or *tt7-2* in Figure 8*B*. These data are consistent with maximal auxin delivery in the absence of exogenous auxin in both *tt4* alleles and *tt7-2* and cannot account for opposite lateral root phenotypes between *tt4* and *tt7* alleles.

## Discussion

Flavonols are a subclass of flavonoid specialized metabolites that function as antioxidants in plants and regulate developmental and stress responses (1, 2, 5). *Arabidopsis* produces three flavonols, kaempferol, quercetin, and isorhamnetin (2, 74), which can be further modified *via* glycosylation, leading to a set of molecules with different antioxidant capabilities and distinct functions *in vivo* (5, 9, 13). The *tt4*(*2YY6*) and *tt4-1* mutants, which produce no flavonols, were previously reported to have increased numbers of lateral roots (18, 58). This study used a genetic approach to determine which flavonol is important for lateral root development and whether these biosynthetic and developmental defects were reversible using genetic and chemical complementation. Furthermore, we asked whether the alteration in root development in flavonol biosynthetic mutants is tied to their role as antioxidants.

Our experiments provide evidence that kaempferol, or a downstream derivative, negatively modulates lateral root development. We examined the lateral root number of a suite of flavonoid biosynthesis mutants that are deficient in all flavonols or produce a subset of flavonols. In mutants that have defects in synthesis of all flavonols (*tt4*-*2*, *tt4*-*11*, and *fls1-3*), there was an increase in lateral root number, which was reversed by genetic and chemical complementation. In contrast, *omt-1* and *tt3*, which have defects in enzyme catalyzing steps in the synthesis of isorhamnetin and anthocyanins, respectively, had wild-type levels of lateral roots. The most striking result was found with two *tt7* mutant alleles, which have defects in the branchpoint enzyme that controls the conversion of the precursor of kaempferol to the precursor of quercetin and had significantly decreased numbers of emerged lateral roots. The lateral root phenotype of the *tt7* mutants, which have elevated kaempferol and no quercetin, suggests that the increased concentration of kaempferol, or a downstream conjugate or metabolite, negatively impacts lateral root development, since the total flavonol level in this mutant is comparable with wild-type. To further establish that kaempferol and its derivatives modulate lateral root development, we treated seedlings with both kaempferol and quercetin. Treatment with either flavonol led to a reduction in numbers of lateral roots in *tt4-2* and *fls1-3*. We also examined the effect of the *tt7* mutation on initiation and emergence of lateral roots and found that this mutation only impairs lateral root emergence. These results are consistent with kaempferol or a downstream metabolite acting as negative modulators of lateral root development. A recent publication found kaempferol can be metabolized to produce ubiquinone, also an antioxidant compound (75) and we cannot eliminate the possibility that higher levels of the products of kaempferol function to modulate ROS and lateral root development.

To understand why there is a reduced level of lateral roots in *tt7*-2, we examined the localization patterns of kaempferol and quercetin using the flavonol dye DPBA. In wild-type kaempferol accumulates in the LRP while quercetin has a more dispersed distribution, both flavonols are absent in *tt4*, while in *tt7*, kaempferol-DPBA fluorescence is increased in lateral root primordia compared with wild-type where it is appropriately localized to impact lateral root development. Using transcriptional and translation fusions (CHS-GFP and FLS-GFP), we observed GFP fluorescence within lateral root primordia consistent with flavonol localization patterns determined using DPBA. The expression of the CHS-GFP fusion was limited in the primordia suggesting that there is transport of precursors into developing LRP. Consistent with this possibility, prior research has shown that naringenin can be transported long distances from the shoot to the root and vice versa, although flavonols do not move between tissues (16, 76). What is evident from these results is that there is precise spatial localization of flavonol accumulation, with different patterns found for quercetin and kaempferol.

We tested the hypothesis that the enhanced lateral root formation in the absence of flavonols was tied to altered auxin transport, but our results do not support this possibility. Mutants that have defects in flavonol synthesis have increased auxin transport in roots in both the shootward and rootward directions (11, 17, 18) and auxin drives lateral root formation (51, 52). We asked whether global or local treatments with IAA resulted in differences in induction of lateral roots that explain the opposite effects of the *tt4* and *tt7* mutations on lateral root numbers. There was an induction of lateral roots in all mutants and wild-type in response to global treatment with IAA, while none of these mutants responded to auxin applied at the root shoot junction to deliver auxin that drives lateral root emergence (72, 73). This treatment increased the number of lateral roots formed in Col-0, but the *tt4*-*2*, *tt4*-*11*, and *tt7-2* mutants showed no significant increases in lateral root numbers. These results are inconsistent with the opposite root developmental patterns in *tt4* and *tt7* being tied to flavonol regulation of auxin response or auxin transport, as the mutants respond similarly to this treatment.

Our results better support the hypothesis that the function of flavonol metabolites is to scavenge ROS that modulates lateral root development. Flavonol compounds have been shown to act as antioxidants *in vivo* to modulate stomatal aperture (10, 14), pollen viability (15), and root hair number (9). To ask if the developmental effects of flavonol mutants were tied to the ROS status, we treated roots with ascorbic acid and NAC. As an antioxidant, ascorbic acid has been shown to scavenge radical species (66) and is involved in reduction of ascorbate peroxidases that scavenge H_2_O_2_ (77). This antioxidant reduced the number of lateral roots in the *tt4* mutants to wild-type levels but had no effect on root development in *tt7-2*, likely due to elevated levels of the flavonol antioxidant, kaempferol, in developing lateral roots. Similarly, NAC is a form of cysteine that is thought to replenish the glutathione pool as well as reduce thioredoxin, peroxiredoxin, and glutathione reductase proteins. These systems most readily scavenge H_2_O_2_ compounds (67, 68). These results suggest that in *tt4* mutants elevated ROS drives the increase in lateral roots, while the increased kaempferol in LRP in *tt7-2* is sufficient to reduce ROS to levels that impair lateral root formation.

We examined ROS levels within the LRP or in tissues through which lateral roots emerge in flavonol mutants to ask whether the flavonol antioxidant activity modulates LR emergence. We visualized patterns of ROS accumulation using a general ROS sensor, DCF, and a superoxide selective sensor, DHE. In wild-type roots, DCF and DHE fluorescence is the highest in roots above the LRP. The *tt4* mutants and *tt7-2* have an increased DCF and DHE fluorescent signal above the LRP compared with wild-type. The elevated signal above the lateral root may be due to the absence of quercetin in this tissue in both mutants. Quercetin is a potent flavonol antioxidant, which in wild-type is distributed throughout the root including in the layers overlying the primordia. The *tt4* mutants and *tt7-2* have the opposite lateral root phenotypes and similar ROS signals above primordia suggesting that the level of ROS in this region does not account for the developmental differences between *tt4* and *tt7-2*.

The accumulation of superoxide within the LRP, as judged by DHE signal, is a more logical target of flavonol-modulated lateral root development. DHE fluorescence, but not DCF, is detected within the LRP and is at reduced level in lateral root primordia of the *tt7-2* mutant, which have elevated kaempferol in primordia, as shown by DPBA fluorescence. Within the LRP, the DHE signal between Col-0 and *tt4-2* was equivalent. The reduction of DHE signal within the LRP of *tt7-2* is consistent with the twofold increases in kaempferol accumulation within primordia as detected by DPBA and across the entire root by LC-MS.

An important question is what leads to the localized superoxide accumulation within lateral root primordia and how does this ROS act to regulate development. Superoxide compounds can be produced by respiratory burst oxidase homologs (RBOHs) and then can be converted to hydrogen peroxide *via* superoxide dismutase (78). RBOHA-F localizes in lateral root primordia determined using a GUS reporter in a prior report that suggests that auxin induces transcription of the RBOH proteins increasing ROS above and within the lateral root primordia promoting initiation and emergence (35). RBOHB and RBOHF localize at the base and the tip of the lateral root primordium, while the other RBOH proteins localize throughout the primordium (35). The RBOH enzymes are therefore appropriately positioned to control the synthesis of superoxide in the developing lateral root primordia. RBOH proteins synthesize superoxide into the apoplast where it is converted to hydrogen peroxide that can enter cells through aquaporins (79). If these proteins are embedded in the membrane facing into the primordia, this could account for elevated superoxide within the primordia.

The superoxide in the LRP may modify lateral root development using several possible mechanisms. This molecule may directly regulate the activity of proteins or mechanical properties of cell wall polymers or superoxide-derived hydrogen peroxide may oxidize cysteine residues on proteins to alter their structure, activity, or function (80). A study identifying proteins that contain an oxidative modification in *Arabidopsis* found that 4% of detected proteins were enriched in cell wall proteins (81). For instance, RBOHF has been shown to regulate lignification of plant cell walls (82). Hydrogen peroxide has been implicated in strengthening of the cell wall through cross-linking of cell wall polymers (83). In contrast, hydroxyl radicals may loosen the cell wall (84). Cell wall structure is integral to lateral root emergence since the LRP must move through multiple cell layers requiring tightly controlled cell wall remodeling and mutants with impaired cell wall modifying enzymes have altered lateral root development (85, 86, 87). The cells within the primordia need to have strong cell wall structure to push through cell layers, and the cells overlying the primordia need to have their cell wall loosened to allow for emergence (85, 87). Therefore, one possible scenario is the decrease in the superoxide pool within the LRP in *tt7-2* may contribute to a less dense cell wall within the LRP impairing lateral root emergence.

The upregulation of flavonol biosynthesis is well described in response to environmental stress, hormones, and development signals (2, 9, 10, 14, 15). Additionally, flavonoid precursors can be transported both short and long distances (64, 65), which could allow a role for flavonols in long distance signaling. Several recent studies have illustrated important roles of long-distance root to shoot signal transport in controlling root development (88). The HY5 transcription factor named for its control of hypocotyl elongation also regulates primary root elongation and lateral root emergence, *via* mediating interactions between light and auxin signaling. Light-dependent synthesis of flavonols is positively regulated by this transcription factor (89). HY5 was suggested to move as a long-distance signal from the shoot to the root, but recent findings suggest HY5 controls synthesis of this mobile signal (88). An intriguing possibility is that HY5-regulated flavonoid precursors produced in the shoot tissue may act as a long-distance signal leading to altered root architecture due to shoot-mediated environmental signals. We focused our study on signaling molecules downstream of flavonols, while signaling molecules upstream of flavonols that lead to root architectural changes are an interesting area of future study.

**Figure 9:**
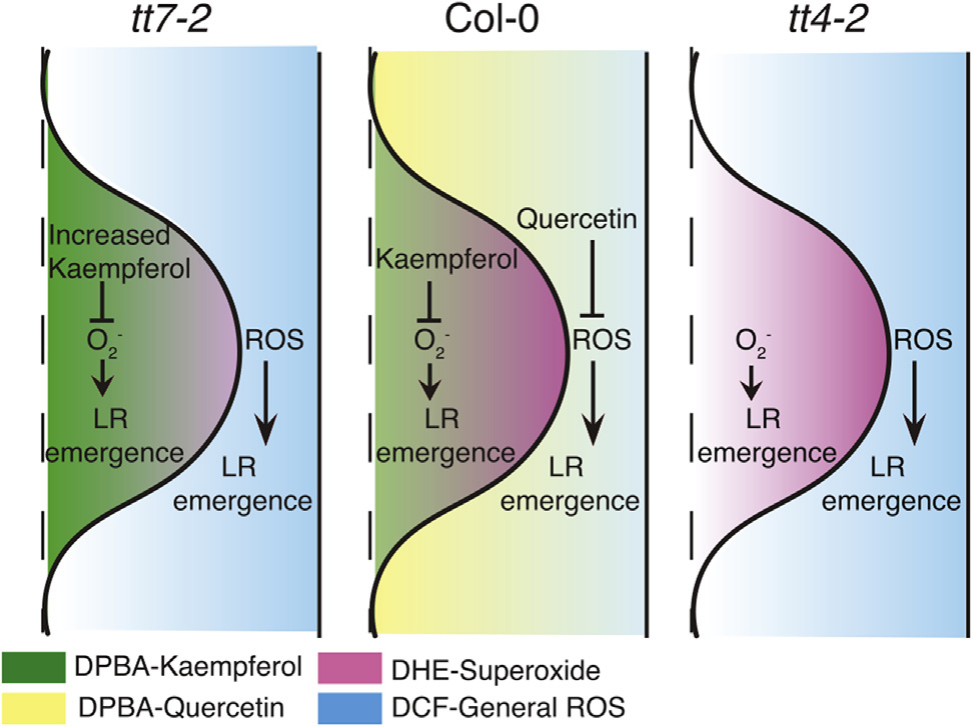
**Kaempferol accumulation with the lateral root primordia reduces superoxide and impairs lateral root formation.** Roots are shown in the order of the number of lateral roots that emerge, with the fewest in *tt7* and the most in *tt4*. Quercetin (*yellow*) is found in the region over the developing primordia and when it is missing in either *tt4* or *tt7*, there is higher levels of DCF-detected ROS (*blue*). This cannot account for the difference in lateral root emergence between these two mutants. In contrast, kaempferol (*green*) accumulates at higher levels in the LRP in *tt7*, and DHE fluorescence (superoxide, *pink*) is lower in *tt7* primordia. Together these findings support the model where the levels of lateral root emergences are driven by superoxide within the primordia and are inversely proportional to the levels of kaempferol with these primordia.

This study shows that flavonol-modulated ROS within the lateral root primordia regulates lateral root emergence and identifies kaempferol and its derivatives as drivers of this response. We constructed a model that summarizes our key findings (Fig. 9). Three roots are shown from left to right in order of the number of lateral roots, with *tt7* forming the smallest and *tt4* forming the largest number of lateral roots. In the absence of flavonols in *tt4*, there is an increase in the number of emerged lateral roots, which can be reversed with genetic and chemical complementation. In contrast, in two *tt7* alleles, which produce elevated levels of kaempferol and no quercetin, there are fewer lateral roots, and this phenotype is linked to the increases in kaempferol. Kaempferol localizes primarily to vascular tissue and the lateral root primordia, and it is elevated in the primordia of *tt7*. The ROS concentration (as judged by DCF fluorescence) was similarly increased in epidermal and cortical cells overlaying the lateral root primordia in the absence of quercetin (*tt4-2* and *tt7-2*). This finding suggests that the ROS accumulation above lateral roots of flavonol biosynthetic mutants does not modulate lateral root emergence. In contrast, the superoxide concentration, as judged by DHE fluorescence within LRP, is lower in *tt7*-2 than either wild-type or *tt4-2*. This model suggests increased kaempferol within the lateral root primordia scavenge superoxide, leading to decreased DHE signal and impairing lateral root emergence. Together these findings suggest that the amount of lateral root formation is inversely proportional to the levels of kaempferol, or its downstream metabolites, and the accumulation of these antioxidant metabolites reduces superoxide that drives lateral root emergence.

## Experimental procedures

### Seeds and growth conditions

*Arabidopsis* mutants used in this study are in the Col-0 background. The following lines been previously published as follows: *tt4-2* (9, 90), *tt4-11* (11, 91), *fls1-3* (76), *tt7-2* (11), *CHS-GFP* (11), *FLS-GFP* (76), *omt-1* (74), and *tt7-1* (75). The *tt3* (SALK_099848C) mutant was obtained from the Arabidopsis Biological Resource Center. The T-DNA insert in the *DFR* gene in the *tt3* mutant was verified by PCR, shown to be the only insert, and homozygous lines were selected.

*A. thaliana* seeds were sterilized in 90 % (v/v) ethanol for 5 min and dried before sowing on 100 x 15 mm petri dishes with 30 ml of 1X Murashige and Skoog (MS) medium (Caisson Labs), pH 5.5, with MS vitamins, 0.8% (w/v) agar (MP Biomedicals), buffered with 0.05% (w/v) MES (RPI), and supplemented with 1.5% (w/v) sucrose. The petri dishes were sealed with micropore tape (3M). Seeds were stratified for 48 h in darkness before being placed in vertical racks under continuous cool white light at 100–120 μmole photons m^−2^s^−1^. After 5 days, seedlings were transferred to either control or treatment plates and allowed to grow for 3 days. Plants treated with IAA and their controls were grown under a light bank with a yellow plexiglass cover to reduce the amount of blue light exposure and reduce degradation of IAA (92). All assays took place 3 days after transfer.

### Lateral root imaging and quantification

Seedlings (8-day-old) were imaged in brightfield mode using a Zeiss Axio Zoom V16 stereomicroscope with a Zeiss AxioCam 506 monochrome camera using panorama mode. Lateral root numbers were quantified in 8-day-old seedlings using an Olympus SZ40 microscope. Statistical analysis of the data was completed in Prism 8 using a two-way ANOVA followed by Tukey's post-hoc test.

For treatments with naringenin, kaempferol, quercetin, N-acetyl-cysteine, or ascorbic acid, seedlings were 5 days old at the time of treatment and lateral root number was quantified 3 days after treatment. MS media was prepared as described above and was cooled for 1 h in a 60 °C water bath after autoclaving before treatments were added to the media. Stock solutions of treatments were prepared for each individual experiment. Naringenin (Indofine Chemicals) was prepared as a 250 mM stock in EtOH and was diluted in the media to a final concentration of 100 μM. Kaempferol and quercetin (Indofine Chemicals) were prepared in 50 mM stocks in ethanol and diluted in the media to a final concentration of 100 μM. N-acetyl-cysteine (Sigma) and ascorbic acid (Sigma) were prepared as a 1 M stock in water and diluted in the media to a final concentration of 1 mM and 500 μM, respectively.

Seedlings treated with IAA were 5 days old at the time of treatment and lateral root number was quantified after 3 days of treatment. MS media was prepared as described previously and cooled for 1 h at 55 °C in a water bath after autoclaving before treatments were added to the media. A stock solution of 10 mM IAA in EtOH was diluted to 100 μM in EtOH and then diluted further within the media to final concentrations of 0.1 μM and 0.3 μM. Treatment with auxin at the root–shoot junction occurred on 5-day-old seedlings transferred to MS media. Agar droplets were made using 0.8% agar and allowed to cool for 20 min. A stock solution of 10 mM IAA was diluted to 100 μM and then diluted further within the agar to final concentration of 1 μM. A pipette was used to measure out 30 μl of the agar with IAA or EtOH and expelled in a droplet on a strip of parafilm. These droplets cooled and solidified for 20 min, before being placed at the root–shoot junction of 5-day-old seedlings. Lateral root number was quantified 4 days after treatment.

### Clearing and quantification of lateral root primordia

To quantify the number of LRP, seedlings were fixed and cleared with the Clearsee method (93). Roots were fixed in 4% (w/v) paraformaldehyde (Acros organics) in PBS (Alfa Aesar) for 1 day. Seedlings were rinsed twice for 1 min in PBS buffer before being transferred to the Clearsee solution (10% xylitol, 15% sodium deoxycholate, 25% urea in water) (w/v). Seedlings were incubated in Clearsee for 2–3 days prior to being mounted on microscope slides in water. The number of lateral root primordia was counted using brightfield mode of a Zeiss Axio Zoom V16 stereomicroscope with a Zeiss AxioCam 506 monochrome camera. The number of lateral roots per seedling are reported. Statistical analysis of the data was completed in Prism 8 using a one-way ANOVA followed by a Dunnett's multiple comparisons test.

LRP were also quantified using a root bending assay. Seedlings were grown to 5 days old and transferred to control plates. The root of each seedling was bent 1–2 mm from the tip to a 90-degree angle. The presence of an LRP or an emerged lateral root was quantified 2 days after bending (48 h). Emerged lateral roots were quantified using an Olympus SZ40 microscope. Seedlings were then imaged under a REVOLVE microscope with a drop of water placed on the bend area to determine if there was a primordium. The formation of a lateral root (emerged or unemerged) is reported in the form of a ratio to the number of roots bent. Statistical analysis of the data was completed in Prism 8 using an ordinary one-way ANOVA followed by a Dunnett's multiple comparisons test.

### DPBA imaging and quantification

Diphenylboric acid 2-aminoethyl ester (DPBA, which is sold under the name 2-Aminoethyl diphenylborinate by Sigma Aldrich; Catalog number D9754) was used to visualize quercetin and kaempferol *in vivo* using previously published methods that use signal differences in emission spectra between the two flavonols to elucidate their unique accumulation patterns (11). Seedlings were stained in darkness in 0.25% w/v DPBA with 0.06% Triton-X (v/v) in water for 12 min, with a 12 min wash in water. Seedlings were mounted on microscope slides in water. Roots were imaged using a Zeiss LSM 880 confocal microscope using the 458 laser at 8% laser power. Images were taken using a 40X objective with an emission spectrum 472–499 for the kaempferol channel and 585–619 for the quercetin channel. Z-stack images were taken to identify the center of the lateral root primordia. All microscope settings were identical for each biological replicate.

DPBA fluorescence was quantified for each lateral root primordia using FIJI. The center of the lateral root primordia was determined, and the channels were pseudo-colored green for kaempferol and yellow for quercetin. A 100-pixel line (which was 11.5 μm) was then drawn from the vascular tissue to the epidermis through the center of the primordia. The fluorescence intensity was measured on average every 0.1 μm along the line and measurements were taken from 0 to 60 μm across the lateral root primordia. A single optical slice was used for quantification and as a representative image.

### *CHSpro:CHS:GFP* and *FLSpro:FLS:GFP* confocal imaging

Eight-day-old seedlings transformed with the *CHSpro:CHS:GFP* (11) or the *FLSpro:FLS:GFP* (76) reporters were imaged using the Zeiss LSM 880 confocal microscope and the 488 nm laser at 12% power and 490–544 emission spectra. Cell walls were stained with 0.5 mg mL^−1^ propidium iodide (PI, Acros Organics) dissolved in water for 5 min. PI was imaged by excitation with a 561 laser with an emission spectrum set to 579–695 nm. All microscope settings were identical for each biological replicate. Z-stacks were taken and compressed into maximum intensity projections using Zen black software.

### DCF and DHE imaging and quantification

General ROS were detected using 2′-7'-dichlorodihydrofluorescein diacetate (CM-H_2_DCFDA, Invitrogen). CM-H_2_DCFDA was dissolved in DMSO to give a concentration of 1 mM and further diluted to a final concentration of 50 μM in water. Both control and ascorbic-acid-treated seedlings were incubated in CM-H-_2_DCFDA for 12 min and washed in water for 1 min in the dark before being mounted on microscope slides in water. Fluorescence was detected using a Zeiss LSM 880 confocal microscope using the 488 nm laser at 5% power with an emission spectrum of 490–606 nm. For each lateral root primordia, z-stack images were generated using the 40X objective with a line averaging of 4. We were careful to minimize photooxidation by using a lower laser power and limiting exposure of the sample to the laser and light. Similarly, we obtained images swiftly after exposure to DCF to limit any photooxidation and changes to the ROS environment that were not related to our experiment.

To verify that the dye was being taken up into the primordia, fluorescein diacetate (FDA, Acros organics) controls were performed. FDA was prepared and imaged in the same manner as CM-H_2_DCFDA. FDA was not taken into primordia of stage 3 and lower, therefore those primordia were not included in the quantification. Zen Blue software was used for quantification of DCF fluorescence. A region of interest was drawn along the epidermis above and below the lateral root primordia. The region of interest varied slightly based on size of the lateral root primordia but averaged to 3185.7 μm^2^ for above the primordia and 3010.1 μm^2^ below the primordia. Statistical analysis of the data was completed in Prism 8 using a two-way ANOVA followed by Tukey's post-hoc test.

Superoxide accumulation was visualized using DHE. DHE was dissolved in DMSO to a concentration of 20 mM and further diluted to 50 μM in water. Eight-day-old seedlings were incubated in DHE for 25 min and then rinsed in water before being mounted on microscope slides. Fluorescence was detected using a Zeiss 880 confocal microscope using the 488 laser at 2% power with an emission spectrum of 490–561 nm. For each lateral root primordia, Z-stacks were generated using the 40x water objective with a line averaging of 4. FIJI was used for image quantification. A 100-pixel line (11.5 μm) was drawn from the vascular tissue to the tip of the lateral root primordia. Statistical analysis was completed in prism 8 using a two-way ANOVA followed by Tukey's post-hoc test.

### Extraction and quantification of aglycone flavonoids by LCMS

Flavonols were extracted as previously described (9). Roots of 7-day-old seedlings were separated from shoots and flash frozen in liquid nitrogen and immediately used for extraction or stored at –80 °C until extraction. The extraction buffer had an internal standard of 500 nM formononetin (Indofine chemicals) that was dissolved in 100% acetone (Optima-grade, Fisher Scientific). The extraction buffer was added to the samples, which ranged between 17.65 mg and 65.01 mg, at 3 μl/mg. Tissues were homogenized using a 1600 MiniG tissue homogenizer (Spex Sample Prep) for 10 min. An equal volume of 2 N HCl was added to the samples and they were incubated at 75 °C for 45 min to produce aglycone flavonols. Three-hundred microliter of ethyl acetate (Optima grade, Fisher) was added to the samples and shaken for 5 min before centrifugation at maximum speed for 10 min. The top organic phase was isolated, and the ethyl acetate phase separation was repeated. The collected organic phases were pooled. The organic phase was dried by airflow using a mini-vap evaporator (Supelco Inc). Samples were resuspended in 300 μl of acetone before LCMS analysis

Samples were analyzed on a Thermo LTQ Orbitrap XL with an electrospray ionization source, coupled to a Thermo Accela 1250 pump and autosampler (Thermo Fisher). For flavonols analysis, 10 μl of each sample was injected with a solvent of water: acetonitrile, both containing 0.1% (v/v) formic acid on a Luna 150 x 3 mm C18 column with a Security guard precolumn. The solvent gradient was 90% water: 10% acetonitrile (v/v) to 10% water: 90% acetonitrile (v/v) in a time span of 18.5 min. From 18.5 min to 20 min, the gradient moves from 10% water: 90% acetonitrile (v/v) back to 90% water: 10% acetonitrile (v/v) and holds at these concentrations for another 2 min to recondition the column. Quantification of flavonols was found by comparing the MS1 peak area data, quantified in Thermo Xcalibur, to standard curves generated using pure standards of naringenin, quercetin, kaempferol, and isorhamnetin (Indofine chemicals). Total flavonol level was calculated by averaging the total flavonol value of each sample. MS2 fragmentation spectra were collected to confirm flavonol identity/structure. Fragmentation was induced using 35-kV collision-induced dissociation and the MS2 spectra of flavonols were compared with standards in Massbank database. MS2 spectra of each flavonol in plant extracts were compared with the MS2 spectra of standards run under our specific conditions. Statistical analysis of the flavonol levels (Table 1) and the ratio of mutants to Col-0 (Fig. 1) were compared between genotypes for each flavonol using in Prism 8 using a one-way ANOVA followed by a Dunnett's multiple comparisons test.

## Data availability

All data are included in this article.

## Conflict of interest

The authors declare that they have no conflicts of interest with the contents of this article.
