# Supplementary material for: Flavonols modulate lateral root emergence by scavenging reactive oxygen species in Arabidopsis thaliana
Source: J Biol Chem. 2020 Dec 25;296:100222. doi: 10.1074/jbc.RA120.014543 (PMC7948594; doi:10.1074/jbc.RA120.014543)
Supplement: Table S1 and S2 and Figures S1 to S5 [file mmc1.pdf]

Flavonols modulate lateral root emergence by scavenging reactive oxygen species in *Arabidopsis thaliana*

Jordan M. Chapman and Gloria K. Muday

Supporting Information
Table S1-S2
Figure S1- Figure S5

**Table S1:** MS verification data of flavonoid compounds			
Flavonoid (Massbank spectra comparison)	m/z (mass per charge)	Retention time (min)	Fragment ions
Naringenin (ML005001)	273.0752	12.52	147.002, 152.91, 163.2, 189.08
Kaempferol (PB004121)	287.0555	12.78	105.08, 111.01, 132.94, 165.03, 212.96
Quercetin (PR306547)	303.0500	11.30	93.04, 120.98, 149.001, 187.02, 201.10
Isorhamnetin (PT104010)	317.0660	12.92	153.03, 229.16, 245.12, 285.02, 302.05

**Table S2: Quantification of flavonoids in roots of 7-day old seedlings by liquid chromatography mass spectrometry in nmole/gfw and abundance is reported relative to the wild-type level.**					
	Naringenin	Kaempferol	Quercetin	Isorhamnetin	Total
tt4-11 (CHS-GFP)	N.D.	0.75 ± 0.30	0.79 ± 0.29	0.72 ± 0.22	0.78 ± 0.28
Statistics were measured by an unpaired t-test between the *tt4-11*(CHS-GFP) samples and Col-0 (there is no statistical differences). N.D indicates values that were not detected because they were below the lowest concentration on the standard curve in both Col-0 and the complemented line.					

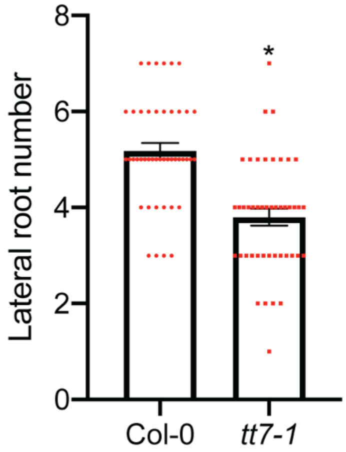

Figure S1: Lateral root numbers are reduced in a second *tt7* mutant. Lateral root number was measured in another *tt7* allele, *tt7-1* over three replicates with an n=45. Significance was determined using an unpaired t-test. (*$p<0.0001$)

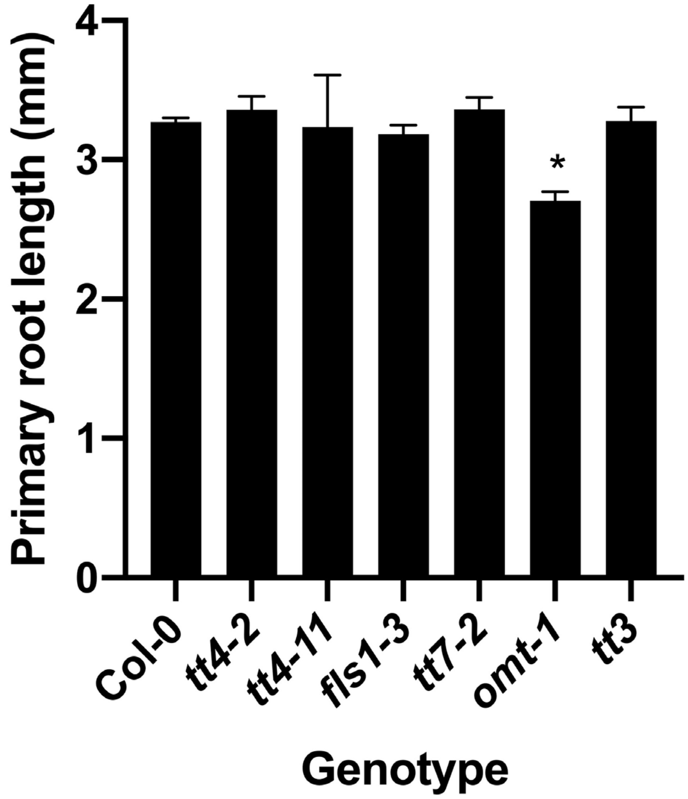

Figure S2: (A) Primary root length is similar in all mutants except *omt-1*, which has significantly shorter primary roots. Primary root length was measured in 8-day old seedlings. Statistics were measured using a one-way ANOVA followed by a Dunnett's multiple comparisons test. (*$p<0.0001$).

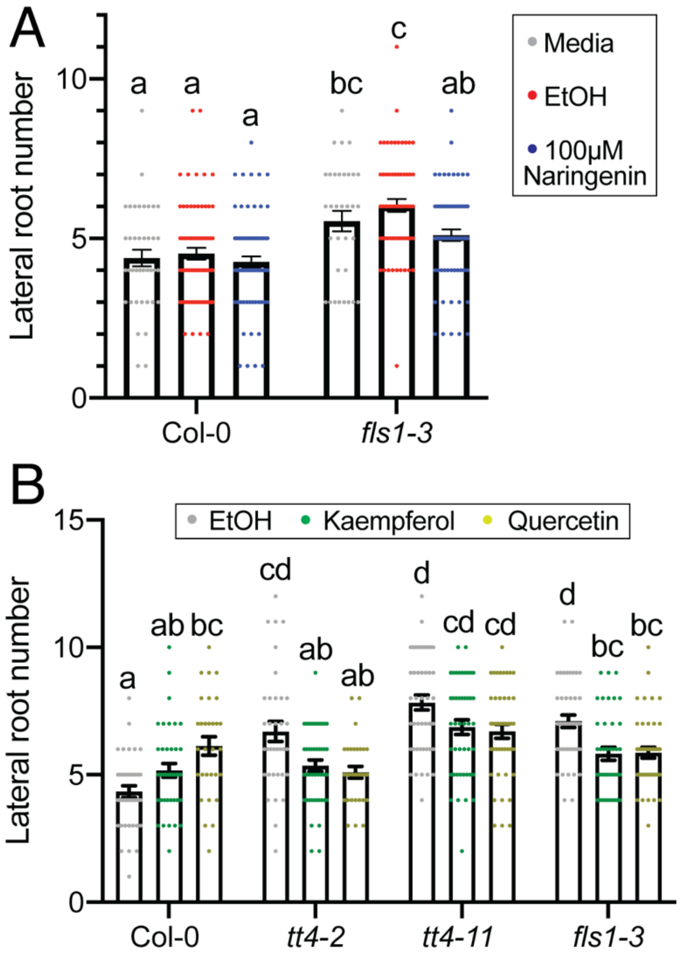

Figure S3: Flavonol treatment reduces lateral root number in flavonol deficient mutants. (A) Lateral root number was evaluated after a three-day treatment with 100 µM naringenin. The naringenin was suspended in ethanol, therefore an ethanol control and a general media control are both reported. Statistics were measured using a two-way ANOVA followed by a Tukey post-hoc test $p < 0.05$. Bars with the same letter represent no statistical difference, while different letters indicate values that are significantly different. (B) Lateral root number was quantified for 8-day old seedlings after a 3-day treatment with either kaempferol or quercetin with four replicates. This gave an n of: Col-0 (control: n=40, kaempferol: n=40, quercetin: n=40), *tt4-2* (control: n=35, kaempferol: n=45, quercetin: n=30), *tt4-11* (control: n=45, kaempferol: n=45, quercetin: n=44), *fls1-3* (control: n=45, kaempferol: n=40, quercetin: n=45). Statistical analyses were performed using a 2-way ANOVA with a Tukey's post-hoc test. Bars with different letters indicate statistical significance.

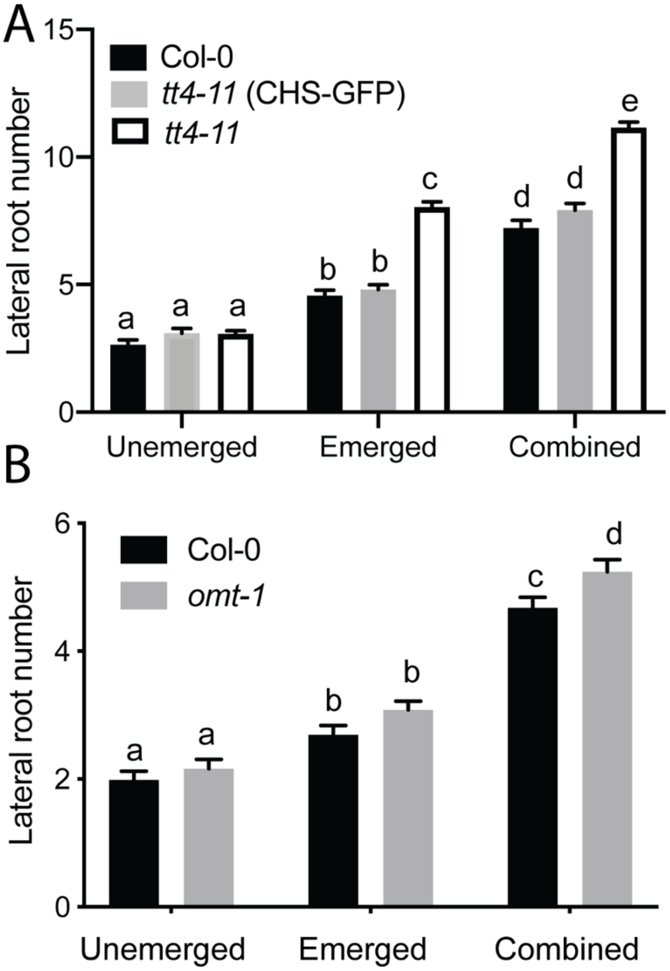

Figure S4: Lateral root emergence is increased in *tt4-11,* but not affected in *omt-1.* (A) The *tt4*-11 mutant has increased emerged lateral roots, but wild-type levels of lateral root primordia. The lateral root primordia (stage4-7) were quantified in cleared 7-day old roots. Statistical differences were determined with a one-way ANOVA. Bars with the same letter represent no statistical difference for each category: unemerged (a), emerged (b-c) and combined (d-e), while different letters indicate values that are significantly different with a $p < 0.05$. (B) Lateral root primordia are comparable between wild type and *omt-1*. The number of lateral primordia (Stages 4-7) and emerged lateral roots, and the combined totals were quantified in cleared 7-day old seedlings. The average and SE from four separate experiments with a total n=60-69. Statistics were determined using an unpaired t-test between genotypes.

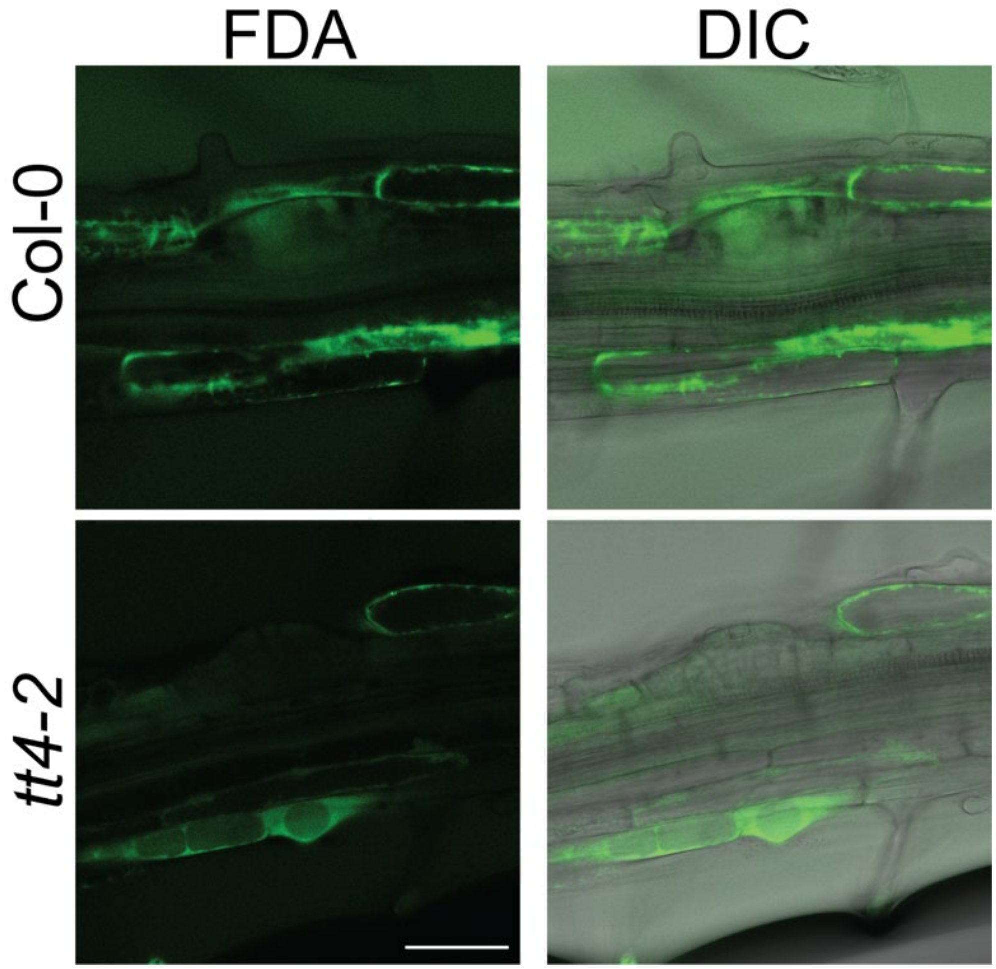

Figure S5: FDA is able to enter later stage lateral root primordia. 8-day old seedlings were stained with FDA and imaged using the same settings across 2 replicates. LRP from stage 2 to stage 8 were evaluated based on their ability to take up FDA. LRP stage 3 and below were unable to take up the FDA. Therefore, experiments using DCF only evaluated stage 4 and higher LRP. Scale bar: 50µm.
